# Quantitative adverse outcome pathway modeling for cigarette smoke-inducible airway mucus hypersecretion. Part 2: Bayesian network modeling for probabilistic risk estimation

**DOI:** 10.3389/ftox.2025.1564864

**Published:** 2025-05-15

**Authors:** Shigeaki Ito, Sakuya Ichikawa, Risa Matsumoto, Shugo Muratani, Keigo Sano, Akina Mori, Kazuo Erami

**Affiliations:** Scientific Product Assessment Center, Japan Tobacco Inc., Yokohama, Kanagawa, Japan

**Keywords:** qAOP modeling, chronic bronchitis, repeated exposure, inhalation, new approach methodology

## Abstract

The development of *in vitro* tests that reproduce real-world situations is crucial for toxicity- and disease-risk assessment without animal testing. Because signs and symptoms of health concerns can be complex, it is helpful to create a simplified representation of such manifestations using a conceptual framework such as an adverse outcome pathway (AOP). Combining an AOP with computational models could be a potential tool for the extrapolation of *in vitro* results to real-world scenarios. Here, we applied Bayesian network-based probabilistic quantitative models for disease-related risk estimation using an *in vitro* dataset on the AOP of mucus hypersecretion—a known representative symptom of chronic airway disease—obtained by repeated exposure of human bronchial epithelial cells to whole cigarette smoke. We also used a computational aerosol dosimetry model to account for differences between *in vitro* exposure concentrations and human exposure scenarios. The results revealed dose- and exposure repetition-dependent increases in adverse outcome probability, suggesting that the model reflects the risk continuum of cigarette smoking. Furthermore, under certain assumptions, dosimetry modeling indicated that our *in vitro* exposure concentrations were similar to actual smoking scenarios. As an exercise, we also calculated *in vitro* odds ratios for chronic bronchitis that were comparable to the range of real-world odds ratios for chronic bronchitis due to cigarette smoking. Our combinatory risk-assessment approach could be a valuable tool for estimating the chronic inhalation effects of inhalable products and chemicals.

## 1 Introduction

The development and use of new approach methodologies for toxicological risk assessments are expected to facilitate the reduction and replacement of animal testing, in line with the 3R (refinement, reduction, and replacement) guiding principles. Recent advancements in such *in vitro* methodologies have enabled researchers to go beyond the cell level by investigating the effects of stressors at the organismal level. Three-dimensionally (3D) cultured *in vitro* cells have the potential to represent tissue- or organ-level complexity with more extended culture duration than monolayer-cultured cells. This culture method presents a significant advantage for assessing the effects of chronic exposure to stressors and toxins. In addition, comprehensive biological phenomena have been elucidated through advancements in omics technologies and systems biology, enabling the identification of key events (KEs) through which certain biological phenomena occur. An adverse outcome pathway (AOP) is a framework that systematically organizes knowledge to depict a simplified theory. For instance, an AOP can be utilized to demonstrate how molecular-level alterations in a specific cell type can lead to apical endpoints in complex organisms, subsuming thousands of intermediate biological processes. The AOP framework is expected to become a useful tool for regulatory decision making.

In Part 1 of this study, we performed an AOP-based study demonstrating that whole cigarette smoke (WCS) induced mucus hypersecretion *in vitro* in 3D-cultured human bronchial epithelial cells (HBECs) (Ichikawa et al.). The AOP structure comprising the following events and assays: molecular initial event (MIE), reactive oxygen species (ROS) generation; KE1, activation of epidermal growth factor receptor (EGFR); KE2, nuclear translocation of specificity protein 1 (SP1); KE3, mucin MUC5AC production; KE4, goblet cell meta/hyperplasia (GCM/H); and adverse outcome (AO), mucus hypersecretion. We also included assays of other key molecules involved in this pathway. Specifically, glutathione (GSH) depletion was assayed because ROS and GSH balance is considered an index of cellular oxidative conditions, and the EGFR ligand amphiregulin (AREG), a modulating factor of the AOP, was assayed because EGFR ligands are crucial for transduction of the EGFR signaling pathway. We modified the AOP structure of a previously reported AOP of “decreased lung function” (Luettich et al., 2017) with practical KEs for the establishment of *in vitro* assays.

Quantitative assessment results at each endpoint were reported in terms of response amplitude in Part 1. However, it is also worth investigating how such results may be connected to actual risk in the real world. Specifically, disease risk is typically described in terms of excess risk ratio or probability. Thus, an AOP-based *in vitro* test dataset alone would be insufficient to provide a quantitative understanding of the risks of stressors in disease-related risk assessment. Several quantitative AOP (qAOP) models have been proposed that bridge the gap between AOP-based *in vitro* test datasets and quantitative requirements of risk assessment. Spinu et al. summarized the existing qAOP models for which conventional statistical, regression, and Bayesian network (BN) modeling approaches were reported (Spinu et al., 2020). Using the qAOP model for chronic toxicity and diseases, Zgheib et al. proposed dynamic BN modeling for chronic kidney disease that can accommodate *in vitro* time-series data (Elias et al., 2019). We also previously developed several qAOP models for chronic effects that allow for consideration of repeated insults (Ito et al., 2024). In the current model, the donor-to-donor variability observed *in vitro* in Part 1 of this study was turned to an advantage, providing an estimation of individual differences following the application of Bayesian-based resampling methods. Such methods have been used in population modeling to estimate parent distribution with limited sub-population data. Bayesian formalisms have been employed in the current model to facilitate calculation of changes in the conditional probability of AO onset over repeated exposures.

Accurate information on actual exposure scenarios is important for realistic risk estimation. Regarding ingested chemical substances, physiologically based pharmacokinetics models based on intravenous injection or dermal absorption can be useful to consider target tissue exposure concentrations (Algharably et al., 2022). In contrast, inhalable substances primarily target the airway, and apical effects can be presented there. To estimate the exposure concentration of a chemical substance during inhalation, a fluid dynamics-based approach is often adopted (Moreau et al., 2024; Daina et al., 2022; Corley et al., 2021; Ramanarayanan et al., 2022). We developed a revised version of the multiple-path particle dosimetry (MPPD) model for aerosols (Mori et al., 2024) that accounts for changes in aerosol droplets and vapor through inhalation. In this study, we adopted the MPPD model for reverse dosimetry of exposure concentration data from Part 1 to investigate the discrepancy between *in vitro* and actual exposure concentrations of WCS.

We report here the first combined quantitative AOP modeling and *in vitro* to *in vivo* extrapolation for the realistic risk estimation of a repeated-exposure scenario. As a proof of concept, we applied this approach to whole cigarette smoke (WCS). Although cigarette smoking is well known as a risk factor for chronic obstructive pulmonary disease (COPD), this approach was developed with a view to applying it also to the risk assessment of other types of tobacco products, inhalable substances, and airborne materials, which may still require animal testing to assess disease risk.

## 2 Methods

### 2.1 Notation convention

As stated in our previous report (Ito et al., 2024), the definitions of mathematical symbols used are as follows. “Exposure repetition is indexed by e=1,2,.,.,E . Donors are denoted by n=1,2,.,.,N . The dose (i.e., exposure concentration) is denoted by d∈D , where D is the set of all doses including non-treatment control. MIEs, KEs, BMs, and AO are collectively referred to as nodes and denoted by v∈V , where V is the set of all nodes. The total number of elements in a set is denoted by |..|; $\left|V\right|$ for example, stands for total number of nodes. Replicates for each donor will be denoted by r=1,2,.,.,R . Matrices are written in double struck upper case letters: $X_{M\times \left|V\right|}$ is a matrix with M rows and $\left|V\right|$ columns. Furthermore, a symbol such as $X^{\left(e\right)}$ means a matrix X for a specific exposure repetition *e*. Similarly, $X^{\left(e,n\right)}$ is understood as a matrix for a specific exposure repetition *e* and a donor n . Elements of a matrix and scalars in general are written in lower-case fonts. A vector is always a column vector and denoted in bold: $\beta \in R^{P}$ is a vector with *P* components. A one vector, $1_{P\times 1}\in R^{P}$ , is a column vector of P ones. Transpose of a matrix or a vector is denoted by the capital letter T in the superscript. A node v is assumed to take continuous values and in Bayesian formalism, the probability that it takes a value *x* depends only on the values of its parents, i.e., $P\left(x^{\left(\nu \right)}\;|\;x\in R^{V}\right)=P\left(x^{\left(\nu \right)}\;|\;x^{\left(\Pi_{v}\right)}\in R^{\left|\Pi_{v}\right|}\right)$ , where $\Pi_{v}$ denotes the set of parents of node v .”

### 2.2 Structure of the AOP

**FIGURE 1:**
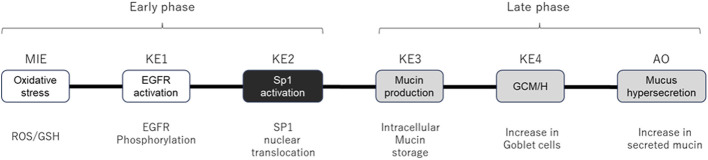
AOP structure for quantitative Bayesian modeling. Schematic diagram of the AOP showing specific assay endpoints for the indicated MIE, KEs, and AO below each event. White and gray boxes indicate early-phase responses and late-phase responses, respectively. KE2 (SP1 activation, black box) was eliminated from the calculation because the response in Part 1 of the study was highly fragile.

We modified a previously reported AOP (Karsta et al., 2017) to facilitate the development of an *in vitro* assay for each KE. Because of the difficulty reproducing and assessing the original “decreased lung function” AOP *in vitro*, we tentatively set mucus hypersecretion as an AO for this study. The modified AOP used in this study comprises the following events: ROS and GSH (MIEs), EGFR activation (KE1), SP1 activation (KE2), mucus production (KE3), GCM/H (KE4), and mucus hypersecretion (AO). The AOP depicted as a directed acyclic graph for Bayesian modeling, is illustrated in Figure 1. Briefly, the assay endpoints of the AOP comprised ROS (MIE), EGFR (KE1), SP1 (KE2), mucus production (KE3), GCM/H (KE4), and mucus hypersecretion (AO), together with GSH depletion and AREG secretion, as the key modulators of the AOP.

### 2.3 Primary dataset

The primary dataset for qAOP modeling was generated from the *in vitro* study of WCS exposure on 3D-HBECs from six different donors, as reported in Part 1 (Ichikawa et al.). Briefly, the assay endpoints of the MIE, KEs, and AO were assessed over time following six exposures to three different concentrations of WCS to observe manifestations of phenotypic changes.

### 2.4 Software and algorithms

Analyses and visualizations were performed using R statistics software version 4.3.2. The R packages used in each analysis are found in our previous report (Ito et al., 2024).

### 2.5 Correlation coefficient analysis and Bayesian resampling of the primary dataset

Correlation coefficients related to each variable were analyzed using our previously reported formulae (Ito et al., 2024). Briefly, the mean fold changes of each assay endpoint related to each donor, dose, and exposure repetition were first log transformed. A vector of the log-fold change $\mu^{\left(n,e,d\right)}\in R^{\left|V\right|}$ was then constructed, where its components $\mu^{\left(n,e,d,\nu \right)},v=1,2,.,\text{and }\left|V\right|$ , were given by the formula $\begin{array}{c} \mu^{\left(n,e,d,v\right)}=\ln\left(\frac{{\left({\bar{f}}^{\left(n,e,d,v\right)}\right)}^{2}}{\sqrt{{\left({\bar{f}}^{\left(n,e,d,v\right)}\right)}^{2}+{\left(s^{\left(n,e,d,v\right)}\right)}^{2}}}\right) \end{array}$ . In addition, the covariance matrix $O_{\left|V\right|\times \left|V\right|}^{\left(n,e,d\right)}$ was calculated using the response-response correlation in the primary dataset, given by the formula $\begin{array}{c} o_{vv^{\prime }}^{\left(n,e,d\right)}={\left(\sigma^{\left(n,e,d,v\right)}\right)}^{2}=\ln\left(1+\frac{{\left(s^{\left(n,e,d,v\right)}\right)}^{2}}{{\left({\bar{f}}^{\left(n,e,d,v\right)}\right)}^{2}}\right)\;for\;v=v^{\prime }\\ o_{vv^{\prime }}^{\left(n,e,d\right)}=\rho_{vv^{\prime }}^{\left(e\right)}\sqrt{o_{vv}^{\left(n,e,d\right)}*o_{v^{\prime }v^{\prime }}^{\left(n,e,d\right)}}\;for\;v\neq v^{\prime } \end{array}$

, to yield the correlation coefficient $\rho_{vv^{\prime }}^{\left(e\right)}=\sigma_{vv^{\prime }}^{\left(e\right)}/\sqrt{\sigma_{vv}^{\left(e\right)}\times \sigma_{v^{\prime }v^{\prime }}^{\left(e\right)}}$ .

Using the equations shown in above, we drew 1,000 samples for each datapoint from a multivariate normal distribution $MN\left(\mu^{\left(n,e,d\right)},O_{\left|V\right|\times \left|V\right|}^{\left(n,e,d\right)}\right).$

### 2.6 Static Bayesian network modeling

As previously reported for our proof-of-concept of qAOP modeling with Bayesian formalisms, we used a Gaussian BN (GBN) represented by a directed acyclic graph defined as the AOP $G=\left(V,E\right)$ , where V is the set of nodes and E is the set of edges between the nodes in V . The directed acyclic graph was topologically ordered such that the parent node(s) was connected to its child(ren) node, where dose was assumed as a continuous variable and included as a root node without parent nodes. The joint probability of each pair of nodes (simultaneous occurrence of both nodes) in a realization $x\in R^{\left|V\right|}$ can be factorized as $P\left(x\right)=\prod_{\nu =1}^{\left|V\right|}P\left(x^{\left(\nu \right)}\;|\;x^{\left(\Pi_{v}\right)}\right)$ , where $\Pi_{\nu }$ is the set of parents of node ν , and $x^{\left(\Pi_{v}\right)}$ is the value that they take. The likelihood $P\left(x^{\left(\nu \right)}\;|\;x^{\left(\Pi_{v}\right)}\right)$ is given by $P\left(x^{\left(\nu \right)}\;|\;x^{\left(\Pi_{v}\right)}\right)=N\left(x^{\left(\nu \right)}|{x^{\left(\Pi_{v}\right)}}^{T}\beta ,\sigma^{2}\right)$ , where unknown parameter β is learned by fitting a linear regression model (Koller and Friedman, 2009; Scutari and Denis, 2014). Fitting between the AOP and $X^{\left(e\right)}$ (data for each exposure) was used as a source of parameter tuning of the model. The probability of activation of each KE was then calculated by resampling from the trained GBNs using a logic-based resampling method, where the probability at each exposure at each dose is denoted as $P\left(KE>\Delta \left|d\in \right.\left[d\pm \varepsilon \right]\right)$ . The dose d is based on the nicotine dosimetry analysis performed in Part 1 of this study. Briefly, the dose in the WCS exposure ranged from 0 to 4,490 μg/mL, which serves as the basis for calculating the dose-probability. Varied threshold values Δ were used to investigate Δ -dependent changes in the probability. To calculate *in vitro* odds ratio (ORs), we used a different approach in which we calculated the fold-change values of the nodes to the air-exposed control at exposure 1.

### 2.7 Dynamic BN modeling

Unlike acute toxic effects, chronic effects manifest after repeated and habitual exposure to a stressor. In addition, because of the complexity of homeostatic mechanisms, biological organisms do not always exhibit the exact same response to stimuli. Therefore, the probability of AO onset with exposure may change over time. To reflect this possibility, we also used dynamic BN modeling to estimate transition probabilities of the AO. As we previously reported (Ito et al., 2024), the upstream events at a previous exposure ( e−τ ) causally influence successive events at the current exposure. The dynamic BN model of the AOP was conditioned using a multivariate Markov process of the form $x^{\left(e,\nu \right)}=X^{\left(e-1,\Pi_{\nu }\right)}\beta^{\left(e,\nu \right)}+\varepsilon^{\left(e,\nu \right)}∼N\left(0,\sigma^{2}I\right)$ . Because the *in vitro* phenotypic changes reported in Part 1 of the study (i.e., mucus production, GCM/H, and mucus hypersecretion) were strongly correlated with one another, as expected, we applied ridge regression to manage overfitting of the model. The appropriate penalty $\lambda^{*}$ was selected using leave-one-out cross-validation. To resample the data from DBM, we used a range from the cutoff value to two times the mean value, and performed likelihood weighing. Like the static BN modeling, we used several activation threshold values for dynamic BN modeling.

### 2.8 Modeling of aerosol deposition in the airway

Deposition of aerosol and chemical constituents in the airway during a puff of the 1R6F combustible reference cigarette was estimated using a revised version of the MPPD model (Mori et al., 2024). While nicotine deposition was used as a representative constituent for this reverse dosimetry approach, the calculation included other representative chemicals. The characteristics of cigarette smoking behavior and aerosol that were utilized in the calculation are summarized in Tables 1, 2, respectively. Briefly, we assumed oral humidity of 80% and lung humidity of 99.9%. The temperatures at the oral interface and lung were set as 20°C and 37°C, respectively. The respiratory tract properties utilized in the deposition estimation are summarized in Table 3. The deposition of specific chemical constituents in cigarette smoke were estimated from the following properties of each constituent: density, vapor pressure, molar mass, specific heat, surface tension, latent heat, activity coefficient, diffusion coefficient in air, diffusion coefficient in H_2_O, and partition coefficient of tissue/air (Table 4). This information was extracted or obtained from Pubchem (PubChem [nih.gov]), ProPhyPlus software, the EPA chemical dashboard (CompTox Chemicals Dashboard (epa.gov), or mathematical calculation, as previously reported (Mori et al., 2024). The surface area of the specific airway loci relevant to human bronchi was estimated from published data (Overton et al., 2001; Weibel, 1979; Weibel, 1963).

**TABLE 1:** User smoking topography utilized in MPPD modeling.

User topography	Input values
Puff withdrawal time(s)	1.62
Mouth-hold time (s)	0.5
Inhalation Time (s)	1
Pause/lung Hold time (s)	0
Exhalation Time (s)	1.62
Puff Volume (mL)	58.5
Inhaled mass(mg)	3.854
Dilution Volume (mL)	400

**TABLE 2:** Aerosol properties utilized in MPPD modeling.

Aerosol properties	Input values
Droplet Number Concentration (×10^9^/cm^3^)	1.0
Droplet Median Diameter (μm)	0.2
Total Mass Inhaled (mg)	3.850
Droplet Temperature (˚C)	24.55

**TABLE 3:** Respiratory tract properties utilized in MPPD modeling.

Respiratory tract properties	Input values
Oral/URT Volume (mL)	50
Functional Residual Capacity (mL)	3,000
Oral Humidity (%)	80
Lung Humidity (%)	99.9
Oral Interface Temperature (˚C)	20
Lung Temperature (˚C)	37

URT, upper respiratory tract.

**TABLE 4:** Properties of cigarette smoke constituents utilized in MPPD modeling.

Constituent	Density	Vapor pressure	Diff Coeff in air	Molar Mass	Diff Coeff in H2O	Latent heat	Specific heat	Activity Coeff	Surf tension	Partition Coeff	Clearance rate	Fraction
	g/cm^3^	kPa	cm^2^/s	g/mol	cm^2^/s	kJ/kg	J/g/K		dyn/cm		1/s	
Water	1.009250	6.330000	0.2000	18.02	0.0000096	36.81	4.200000	1.02629	70.34	233045188905.700000	0.000100	0.7486
Nicotine	0.990059	0.043400	0.0670	162.23	0.0000096	2,414.05	4.177000	1.84998	27.00	22139.739659	0.000100	0.0913
Propylene glycol	1.023380	0.049000	0.1030	76.09	0.0000096	871.65	2.578000	1.99576	34.33	545847.197167	0.000100	0.0379
Glycerine	1.250260	0.000090	0.0958	92.09	0.0000096	961.42	2.471000	0.73771	62.25	347536324.256485	0.000100	0.0779
Triacetin	1.143000	0.001141	0.0605	218.20	0.0000096	360.97	1.694410	119.35900	34.88	511445.020496	0.000100	0.0363
Toluene	0.853000	6.860000	110.6000	92.14	0.0000096	404.99	1.733000	1,327.94000	26.50	13.760748	0.000100	0.0037
Hydroquinone	1.297240	0.000154	0.0857	110.11	0.0000096	747.49	2.098160	0.06011	55.61	8819905.873238	0.000100	0.0020
Catechol	1.220000	0.009700	0.0861	110.11	0.0000096	576.28	1.400000	0.06011	44.30	83113.753584	0.000100	0.0023

## 3 Results and discussion

### 3.1 Dose response of resampled data

**FIGURE 2:**
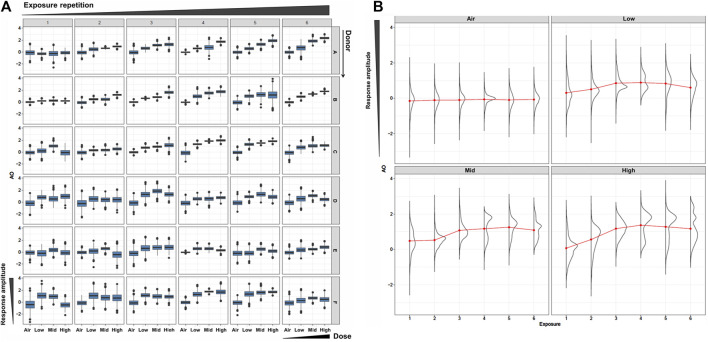
Resampled AO data. **(A)** Box plots of the resampled AO (mucus hypersecretion) data for each donor (vertical) and each WCS exposure repetition (orizontal). The x- and y-axis in each box is for the dose and the response amplitude. **(B)** Density plots of AO results using the combined all-donor resampled data at each dose and exposure repetition.

To robustly calculate AO probability, we first generated an extended dataset using a Bayesian resampling method. This method utilizes distribution of an original *in vitro* dataset to generate a large number of virtual samples. In this study, we drew 1,000 realizations for each datapoint (i.e., dose, donor, and exposure repetition), which we considered robust enough for the probability calculation. As expected, the virtually generated dataset mirrored the dose-response relationship of the original *in vitro* dataset (Figure 2 and see also Part 1 of this study). Using the mean values of the virtually generated dataset, there was no onset of the mucus hypersecretion AO in any donor at the WCS exposure repetition 1. The onset of maximum mucin release varied among the donors, with Donors 1 and 2 showing maximum release at exposure 6, and other donors reaching the maximum at exposures 3 or 4. Additionally, the amplitude of mucus hypersecretion also varied among the donors, with notably less intensive mucus hypersecretion with each exposure over time in Donor 6. While these trends align well with the original *in vitro* data, the virtually generated dataset includes more intense datapoints. Our *in vitro* dataset comprised six donors with three replicates each for all datapoints. As we showed in Part 1, the anticipated donor-to-donor variation was observed, and could be misleading if probability were to be calculated with such a sparse distribution. Other non-Bayesian resampling methods could be employed to complement this sparse original dataset for better estimation of the distribution, thus supporting subsequent modeling efforts. Indeed, resampling methods are helpful with mathematical modeling using *in vitro* data with limited specific endpoints. Furthermore, intense data generation from actual experiments can be time-consuming and costly.

### 3.2 Static BN modeling

**FIGURE 3:**
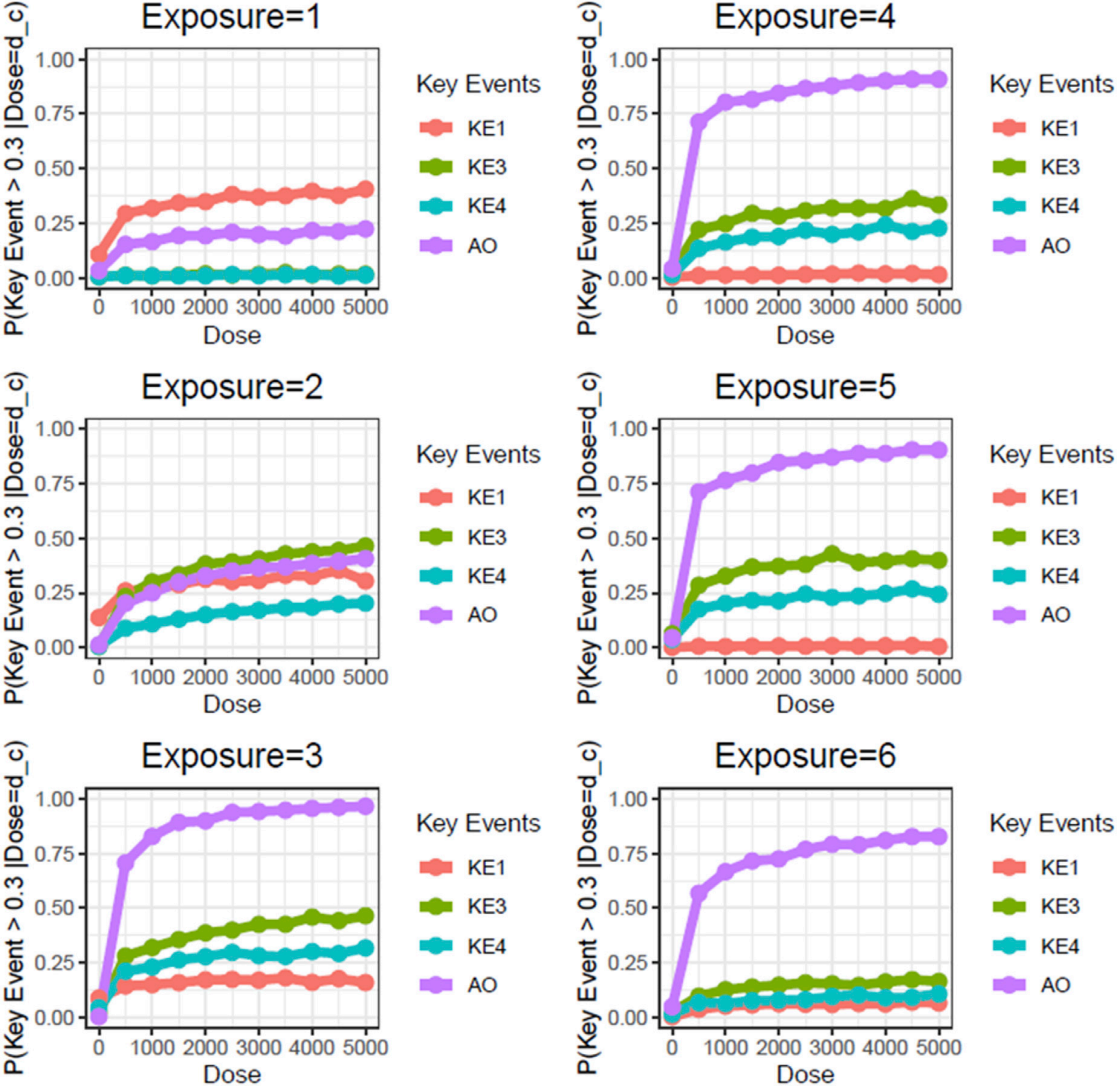
Static BN modeling with the resampled *in vitro* dataset at a threshold of FC = 2.0. BN modeling-based probability calculations of the biological events of the AOP using FC values relative to the air-exposure control at each exposure repetition. The dose was used as the root node. The activation threshold was set as 2.0 (10^0.301).

**FIGURE 4:**
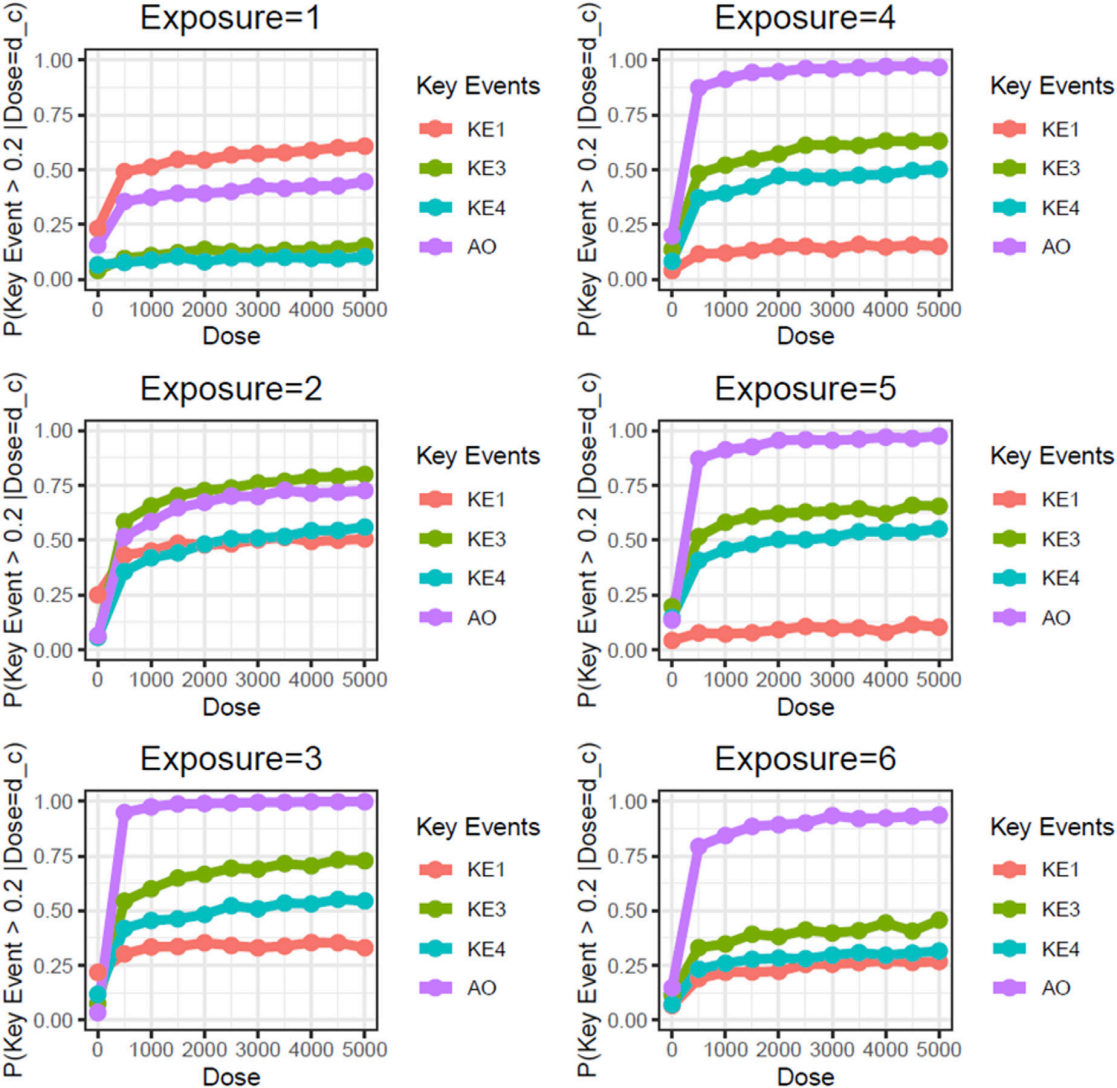
Static BN modeling with the resampled *in vitro* dataset at a threshold of FC = 1.5. BN modeling-based probability calculations of the biological events of the AOP using FC values relative to the air-exposure control at each exposure repetition. The dose was used as the root node. The activation threshold was set as 1.5 (10^0.176).

**FIGURE 5:**
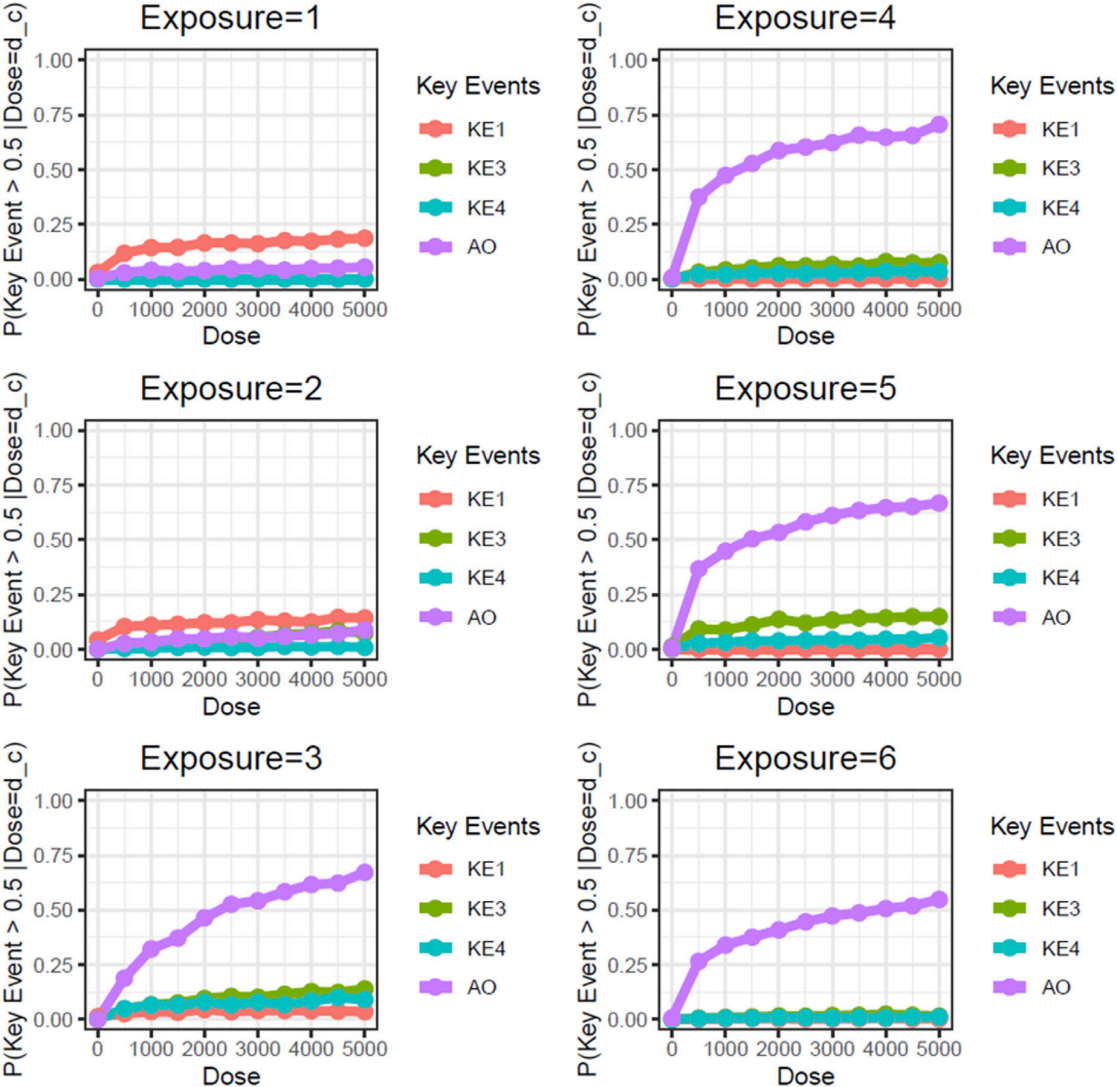
Static BN modeling with the resampled *in vitro* dataset at a threshold of FC = 3.0. BN modeling-based probability calculations of the biological events of the AOP using FC values relative to the air-exposure control at each exposure repetition. The dose was used as the root node. The activation threshold was set as 3.0 (10^0.478).

Next, we performed static BN modeling in which the GBN was separately fitted to the virtually generated dataset for each WCS exposure. Of note, we eliminated KE2 (SP1 activation) from this modeling because of its high variability and large deviation in range of amplitude compared with the other assayed factors. To verify the goodness of model fit, we analyzed the correlation between the predicted probability of each biomarker and the actual observed response amplitude (Supplementary Figure S13). We confirmed that the goodness of fit (i.e., R^2^ values) for KE4 and AO was mostly greater than 0.6, respectively. It therefore suggests that the model adequately explains the relationship between probabilities and response amplitudes, albeit with some degree of uncertainty. Figure 3 shows the probabilities of the KEs and AO calculated at a given dose, where the probabilities were given by *P* (*KE *> Δ∣*d*∈[*d* ± *ϵ*]). For our first attempt, we applied a threshold of fold change (FC) = 2 relative to the control for filtering individual samples, which is typical for *in vitro* testing. The probabilities of all nodes in the AOP showed dose-related increases in the probability at all exposure slices. At the individual KE level, KE1 showed a gradual decrease in probability dependent on the number of exposures, consistent with the change in the amplitude of KE1 over time with each exposure (see also Figure 4 of Part 1). Meanwhile the phenotypic changes related to KE3, KE4, and the AO all showed increased probabilities from exposure 2 onward. However, the probability of the AO was drastically increased from exposure 3 onward compared with those of KE3 and KE4. We also performed probability calculations using the static BN model at several different thresholds. When we loosened the activation threshold from FC = 2 to FC = 1.5, the calculated probabilities of all events were increased, as expected (Figure 4). At an activation threshold set to FC = 3, the probabilities of KE3 and KE4 dropped to approximately zero, while that of the AO remained >0.5 (Figure 5).

Unlike the AO (mucin hypersecretion), KE3 (intracellular mucin production) and KE4 (GCM/H) are directly related to histological changes. These types of biological events are typically limited with regard to response amplitude because changes in a cell population in a limited area are usually represented as a proportion. For example, the proportion of goblet cells in 3D-HBECs cultivated under control conditions ranged from 5% to 10% across all donors, indicating that we might expect maximum increases in GCM/H of 10- to 20-fold relative to control in WCS-exposed cells. Considering that phenotypic changes are not as drastic as disease symptoms, and that basal cells can be retained amidst GCM/H conditions (Polosukhin et al., 2011), the actual response amplitude may be smaller than the theoretical maximum change (i.e., complete conversion of epithelial cells into goblet cells). Although a constant threshold was applied across all of the biological events used in this study, theoretical maximum and minimum changes of each event should be considered in light of the threshold of individual KEs used in the probability calculation.

### 3.3 Dynamic BN modeling

**FIGURE 6:**
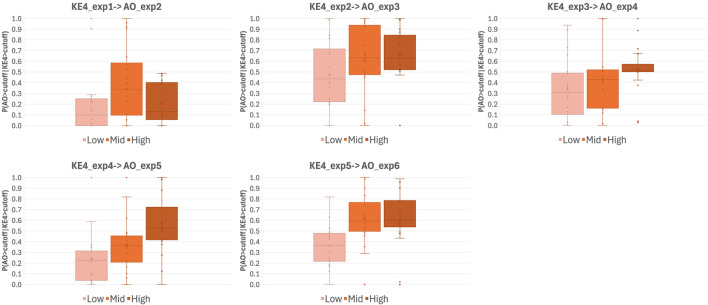
Transition probabilities of AO onset over repeated WCS exposures. Whisker-box plots of transition probabilities of AO onset calculated using a virtually generated dataset with dynamic BN modeling. Probabilities were calculated by extracting all possible combinations of three of the six donors (20 combinations in total) and assuming the activation/onset of KEs and AO had occurred when the log2FC of the response relative to the air-exposure control was >2. Exp; exposure repetition, KE; key event, AO; adverse outcome.

Unlike static BN modeling, dynamic BN captures the influences of previous exposure on the current exposure. Our *in vitro* study reported in Part 1 of this manuscript comprised six repeated WCS exposures, for which we assumed that biological perturbation at the previous exposure could affect the outcome of the current exposure. Indeed, our previous reports showing that repeated exposure to cigarette smoke elicited cumulative inflammatory effects on 3D-HBECs (Ito et al., 2018; Ishikawa and Ito, 2017) imply that such cumulative impacts may stem from biological perturbations that surpass the regulatory capacity of homeostatic processes. These findings also suggest that toxicity, as well as disease onset manifested by chronic exposure to stimuli, can be reproduced in an *in vitro* system. Considering that an insult caused by an early exposure may inform the eventual damage, contextual analysis over repeat exposures is crucial to estimate the risk of a stimulus. Dynamic BN modeling is capable of capturing the relationship between time-separated exposure slices as a transition probability at a given threshold. Similar to the static BN modeling, we used virtually generated data based on the *in vitro* dataset obtained in Part 1 of this study for dynamic BN modeling. First, we calculated the transition probability of the AO (mucus hypersecretion) with the distribution given by all-donor means and standard deviations, at a threshold of log2FC = 2, revealing an almost clear dose-probability relationship (Figure 6).

**FIGURE 7:**
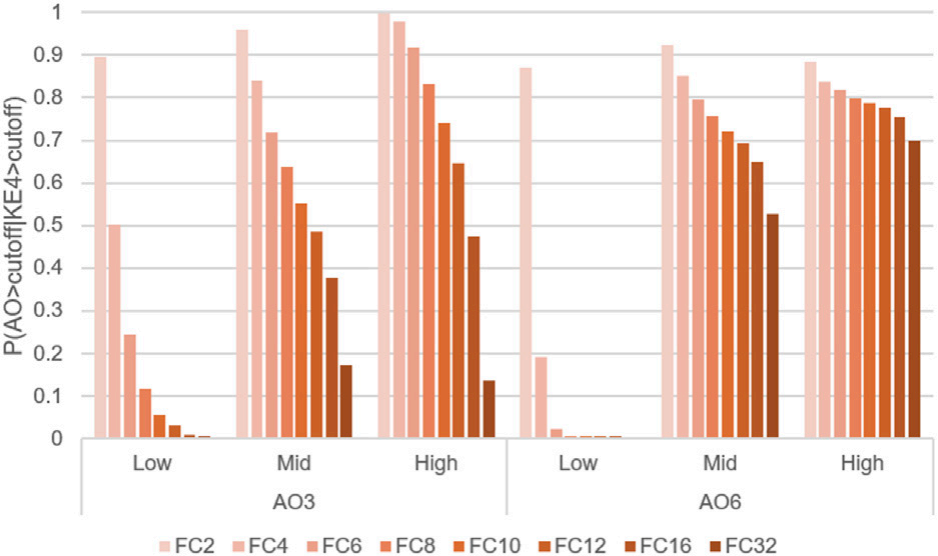
Comparison of transition probabilities P(AO|KE4) at WCS exposures 3 and 6, using different activation thresholds. Probabilities of transitioning from KE4 to AO under three doses of WCS at exposures 3 (AO_3) or 6 (AO_6), calculated under activation thresholds defined as fold change values = 2–32 relative to the air-exposure control value.

Regarding AO transition probabilities at the highest WCS dose, the maximum probability was detected at exposure 3, declining from exposure 3 to exposure 4, and rising again at exposure 6. This result may reflect the donor-to-donor variability in timing at which the maximum response amplitude was observed (see also Part 1 Figure 7). Most donors showed a clear dose response from exposure 3 onward, while Donors 2, 4, and 5 showed a maximum amplitude at exposure 3 (Figure 7C of Part 1). Likewise, some fluctuation in the AO response amplitude over time was observed in all donors except Donor 1, who exhibited a monotonic increase in the response to repeated exposure over time. This was clearly reflected in the distributions of combined all-donor dose-response data, which showed two peaks at exposure 4 onward (Figure 2B), which implies responsive population and non-responsive population.

To evaluate the influence of threshold setting, we also calculated AO transition probabilities at a variety of thresholds. Figure 7 illustrates a comparative analysis of the probabilities of AO at exposures 3 and 6, with a threshold of fold change values = 2–32. As expected, the probability of AO declined as the threshold was raised (Figure 7).

However, there were obvious differences in AO transition probability between exposures 3 and 6. A more pronounced decrease in AO probability was observed at exposure 3, with a moderate influence of the threshold setting at exposure 6. When looking at the actual *in vitro* data shown in Part 1, all-donor mean of the response amplitude of AO at the highest dose at exposure 6 was more pronounced than that at exposure 3 while the increase in KE4 was comparable. Because the probability calculation is influenced by the closest upstream biological event (KE4 in this case), these results suggested that early activation of KE4 (GCM/H) may not directly lead to a robust increase in the AO (mucus hypersecretion). Although goblet cells are a source of mucin, its release from goblet cells is triggered by various stimuli, including cigarette smoke (Cao et al., 2018; Gray et al., 2004; Jaramillo et al., 2018; Zou and Hastie, 2005), regardless of GCM/H. Therefore, the probability increases at exposure 3 might reflect a mixture of acute mucin release and the cumulative effects of WCS insults. Meanwhile, the AO transition probability at exposure 6 was stably high, even at the strictest threshold. The mean values of KE4 at exposures 2 and 5 at the high dose of WCS were comparable (Figure 5B in Part 1); however, the distributions showed different trends. The distribution of KE4 under the high dose at exposure 5 could be divided into two distinct populations: responsive and non-responsive (Supplementary Figure S1). The responsive population could have contributed to the robust AO transition probability at exposure 6. From a biological perspective, this would also mean that the mucus hypersecretion observed at exposure 6 was mainly caused by mucin released under GCM/H conditions.

### 3.4 Linking *in vitro* exposure conditions with real-life cigarette usage

In this reverse dosimetry study, we used nicotine concentration as a representative aerosol constituent. As shown in Part 1, the *in vitro* nicotine concentrations through WCS at low, medium, and high exposure doses were 0.51, 1.62, and 4.49 μg/mL, respectively. Converting these figures to concentration per tissue surface area using the 110 μL collection volume of nicotine and the 0.33 cm^2^ surface area of the culture insert yielded approximate exposure concentrations of 0.17, 0.54, and 1.50 μg/cigarette/cm^2^/day, respectively. We previously reported a revised MPPD model that allows estimation of the deposition fraction of a complex aerosol such as cigarette smoke (Mori et al., 2024). This model is capable of estimating the deposition of specific chemical constituents in the respiratory tract on a cell generation-by-generation basis. Here, we utilized this model to compare the exposure concentrations between the experimental conditions and asymmetric lung geometry data from a previous study of real-life cigarette use including the oral compartment (Mori et al., 2024). The user topography, aerosol properties, respiratory tract properties, and constituent properties are summarized in Table 1 through 4, respectively. We calculated the total surface area of the Trachea-Bronchi region using the method described by Overton et al. (2001). Because it was reported that no goblet cells are found at the 7^th^ generations or deeper (https://ntp.niehs.nih.gov/atlas/nnl/respiratory-system/lung/MetaplasiaGobletCell), we summed the surface areas of generations 0 to 6. The calculated surface area of the TB region was 168.07 cm^2^, equivalent to a nicotine deposition concentration in the actual use scenario of 1.46 μg/cm^2^/cigarette. Thus, the range of nicotine deposition in the *in vitro* exposure conditions and the actual use scenario of one cigarette were comparable.

### 3.5 Biological significance and *in vitro* to *in vivo* extrapolation

Considering the contrast between the duration and frequency of WCS exposure *in vitro* (equivalent to one cigarette a day, three times a week for 2 weeks) with that of real-world smoking (20 cigarettes a day for decades), the lifetime total exposure in these two scenarios are different. Based on a report by Tsuji et al. (2013), an equivalent daily exposure dose should induce equivalent pulmonary responses; however, we observed mucus hypersecretion and the raised probability of this AO under *in vitro* conditions within 2 weeks of exposure. Therefore, there may still be gaps between *in vitro* 3D- HBECs and actual lung tissues. One possible explanation for this discrepancy derives from the disparity in lifespan between actual tissues and those cultivated *in vitro*. While one of the commercially available 3D-HBEC models boasts a shelf life of up to 1 year (https://www.epithelix.com/), actual bronchial tissues retain their function for up to 100 years. Differences in homeostatic capacity, including tissue repair and regeneration, could also contribute to this gap. Various non-epithelial cell types are involved in the maintenance of lung tissues (Lloyd and Marsland, 2017). For example, fibroblasts, immune cells, and epithelial cells orchestrate the repair of damaged tissue; however, this process is a double-edged sword, because such repair sometimes fails and causes unexpected tissue remodeling (Ishida et al., 2023). Given the difficulty in reproducing all homeostatic processes *in vitro*, extrapolation of the factors involved in longer lifespan of *in vivo* tissues to 3D-cultured cells *in vitro* could be crucially important for the further development of *in vitro* models.

**FIGURE 8:**
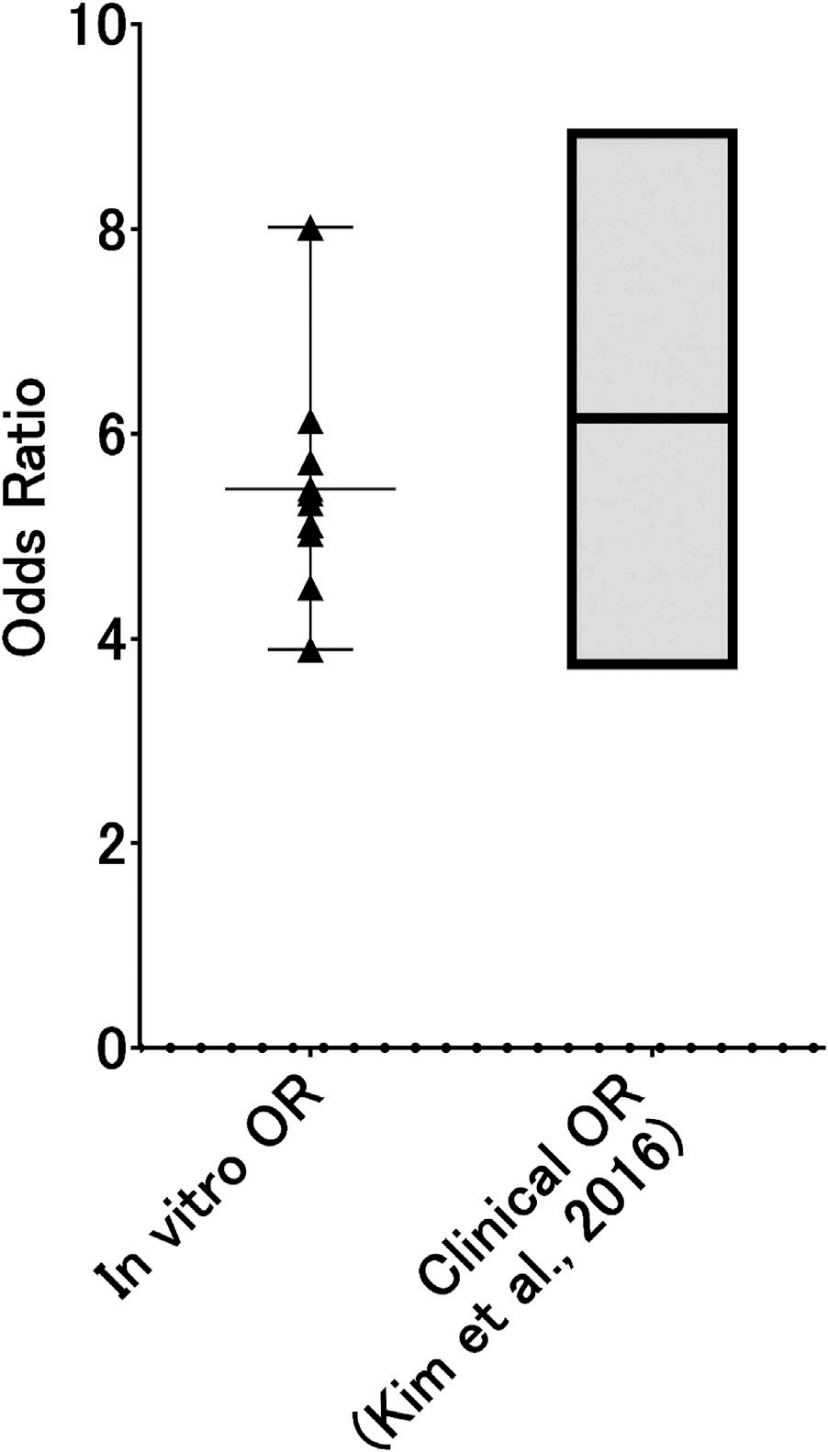
*In vitro* ORs of GCM/H as a function of WCS dose. Probabilities of GCM/H calculated using a static BN model in which the air-exposure control at exposure 1 was used as the absolute control were used to calculate *in vitro* ORs at exposure 6. The dots represent the *in vitro* OR at each inferred exposure dose, where the gray area indicates the range of ORs of real-world chronic bronchitis with and without habitual cigarette smoking (i.e., the ORs for persistent smoking).

Assuming that *in vitro* 3D-HBECs can be utilized to capture the symptoms of respiratory diseases as a form of accelerated study, the activation thresholds should be aligned with the corresponding real-world situations. Mean International Organization for Standardization (ISO) tar values of smokers in eight different countries were reported to range from 5.1 to 10.2 mg tar/cigarette (Mariner et al., 2011). Because we used reference cigarettes (ISO tar value of ∼10 mg) in Part 1 and the deposition simulation in this study, the calculated probabilities of AO onset may be directly applicable in this *in vitro* to *in vivo* extrapolation approach. In addition, based on a previous report by Radicioni et al. (2021), the absolute concentration of MUC5AC is approximately 4-fold higher in current or former smokers without COPD, and approximately 10-fold higher in those with severe COPD, compared with that in non-smokers without COPD, as represented by fold changes in median values. Based on this information, we calculated the OR from the results of static BN modeling at a comparable dose (the medium dose of WCS) with the activation threshold of 10. Because both mucus secretion and GCM/H gradually increased in 3D-HBECs even under control conditions (Figure 6 of Part 1), we designed an exercise to test whether this reflects an aging effect. Considering exposures 1 and 6 as the initiation of smoking and the time point of disease manifestation, respectively, the changes in biological events on the AOP could then be calculated as the fold change relative to the air-exposed control at exposure 1 at dose 1. We were then able to calculate the probabilities of each KE and the AO, even in the control condition, making it possible to also calculate the ORs. Because GCH/M is a representative symptom for chronic bronchitis, we compared the calculated ORs of GCM/H *in vitro* with those from real-world patients with chronic bronchitis. As expected, the ORs of GCM/H for WCS exposure were nearly zero at exposures 1 and 2, gradually increasing with additional exposure to ultimately reach ∼4.5 at comparable nicotine concentration of approximately 1,500 μg/mL (Figure 8). Additionally, because our BN modeling was able to infer the dose-probability relationship as a continuous value, we calculated the ORs of GCM/H for different nicotine concentrations (500–5,000 μg/mL), which ranged from 3.89 to 8.02. The calculated *in vitro* ORs showed good agreement with the established 95% confidence interval of the OR of persistent smoking for chronic bronchitis (3.702–8.983) (Kim et al., 2016). Although the agreement between the *in vitro* ORs and the real-world OR could be coincidental, as the real-world disease manifestation involves some confounding factors such as air pollution (Wang et al., 2022), which was not reflected in our *in vitro* test method. Therefore, the *in vitro* OR may not represent absolute risk of cigarette smoking. However, we believe that calculation of *in vitro* OR under the consideration of realistic exposure scenario would be one of the ways to assess risk of disease as well as chronic toxicity without animal testing.

Notably, we modified the original AOP to align with the feasibility of *in vitro* assays. The original AO, lung function decrease, was replaced with mucus hypersecretion. This change was made because the 3D-HBEC model does not represent the entire lung and is therefore unsuitable for evaluating lung functionality. Consequently, we used GCM/H as a representative condition of chronic bronchitis. However, decreased lung function is a hallmark of chronic disease symptoms and should be replicated *in vitro*. We believe that advancements in in vitro cell culture technology will eventually allow us to evaluate adverse outcomes as accurately as *in vivo*, thereby enabling more precise disease risk assessments.

## 4 Study limitations

Although we have demonstrated that BN-based qAOP modeling could be useful for risk estimation, there are some potentially limiting aspects of our study. First, we used *in vitro* data from six different donors; however, further investigation to determine the ideal number of donors and its potential impact on the probability calculations. Second, because the *in vitro* data were derived from primary HBECs with limited proliferation, once these cells are depleted, cells from different donors will need to be used. This could result in change in the probability calculation, because primary cells may retain original donor-specific characteristics, the response to stimuli may vary from donor to donor. Third, the sustainability and robustness of this qAOP framework require further consideration. We set a uniform threshold across all biological events on the AOP due to the lack of consensus on activation thresholds. However, activation thresholds should vary depending on the biological response, otherwise responses which did not reach the threshold value can be ignored, even though they may still have biological significance. Therefore, it is necessary to explore individual threshold settings or consider normalization as well as regularization for each biological response. Fourth, as discussed above, the longevity of *in vitro* cell cultures differs from that of human tissues and organs. Therefore, it is unknown what duration of real-world smoking corresponds to a 2-week exposure in *in vitro* testing. Further investigation should be conducted to accurately scale the duration of *in vitro* exposure. Fifth, although we used a constant activation threshold across all nodes in the AOP, the actual thresholds may vary from node to node. For example, the elicitation of KE1 may require one threshold to activate the MIE, but a different threshold to activate downstream events.

## 5 Conclusion

Here, we demonstrated the utility of the qAOP model using mucus hypersecretion caused by cigarette smoke as a case study. Although further investigation to verify the relevance of these results for real-world situations is warranted, the combination of *in vitro* repeated exposure and qAOP modeling presented in this series of manuscript would be a powerful tool for comparative assessment with other types of tobacco, including heated tobacco products and e-cigarettes. Additionally, the qAOP models utilized in this study are applicable to other *in vitro* testing scenarios involving repeated exposure, and would enable probabilistic risk estimation for chemicals associated with recurrent insult. We anticipate that our risk estimation framework with qAOP modeling will contribute to ongoing efforts to reduce animal testing, particularly in studies involving repeat exposure.

## Data Availability

The raw data supporting the conclusions of this article will be made available by the authors, without undue reservation.

## Abbreviations

3D-HBECs, Three-dimensionally cultured human bronchial epithelial cells; ALI, Air liquid interface; AO, Adverse outcome; AOP, Adverse outcome pathway; AREG, Amphiregulin; BN, Bayesian network; CSE, Cigarette smoke extract; GCH, Goblet cell hyperplasia; GCM, Goblet cell metaplasia; KE, Key event; MPPD, Multiple-path particle dosimetry; SP1, Specificity protein 1; MIE, Molecular initiating event; WCS, Whole cigarette smoke.
