# Supplementary material for: Quantitative adverse outcome pathway modeling for cigarette smoke-inducible airway mucus hypersecretion. Part 2: Bayesian network modeling for probabilistic risk estimation
Source: Front Toxicol. 2025 May 15;7:1564864. doi: 10.3389/ftox.2025.1564864 (PMC12119607; doi:10.3389/ftox.2025.1564864)
Supplement: Supplementary file 1 [file Presentation1.pptx]

## Slide 1
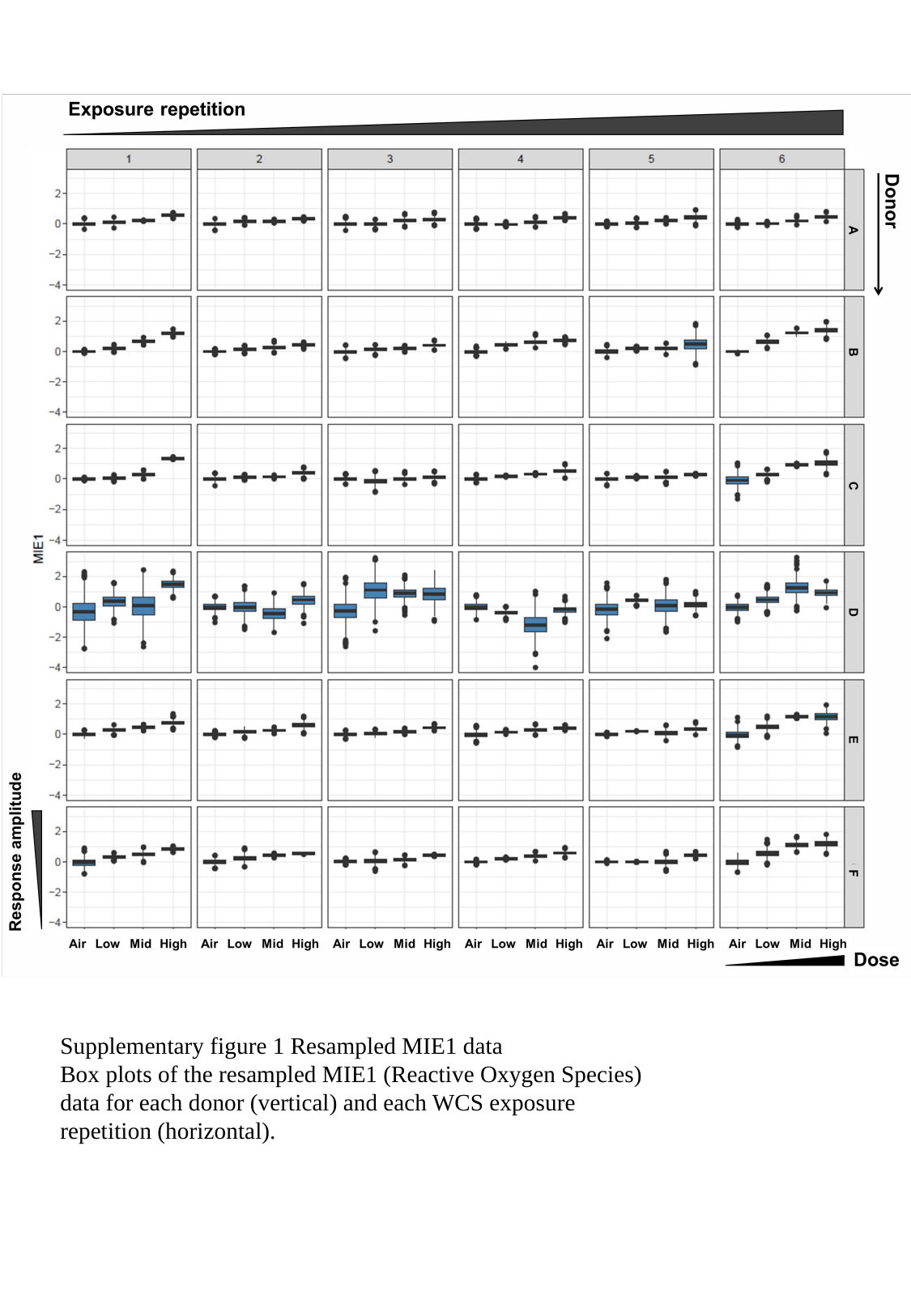

Supplementary figure 1 Resampled MIE1 data
Box plots of the resampled MIE1 (Reactive Oxygen Species) data for each donor (vertical) and each WCS exposure repetition (horizontal).

## Slide 2
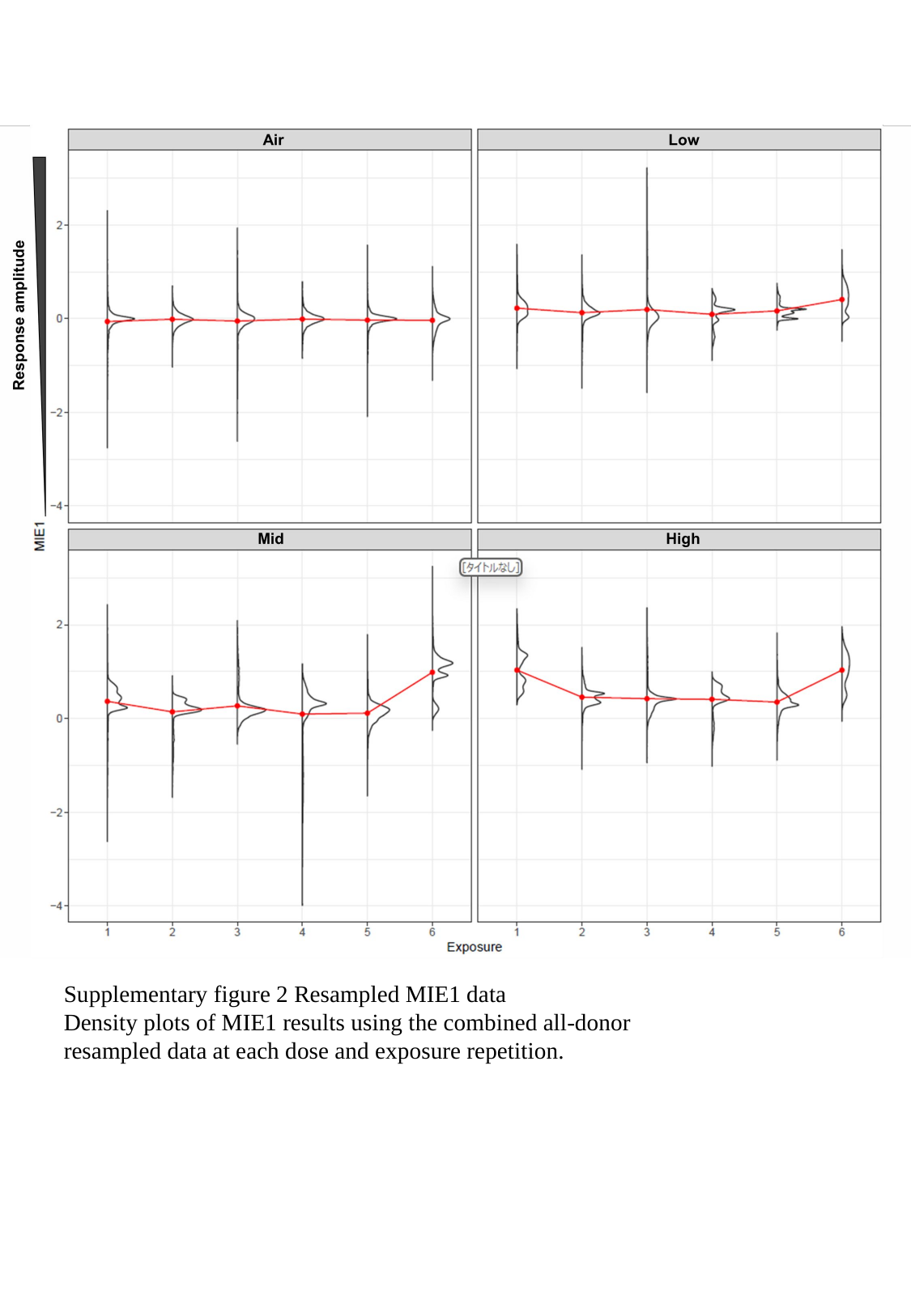

Supplementary figure 2 Resampled MIE1 data
Density plots of MIE1 results using the combined all-donor resampled data at each dose and exposure repetition.

## Slide 3
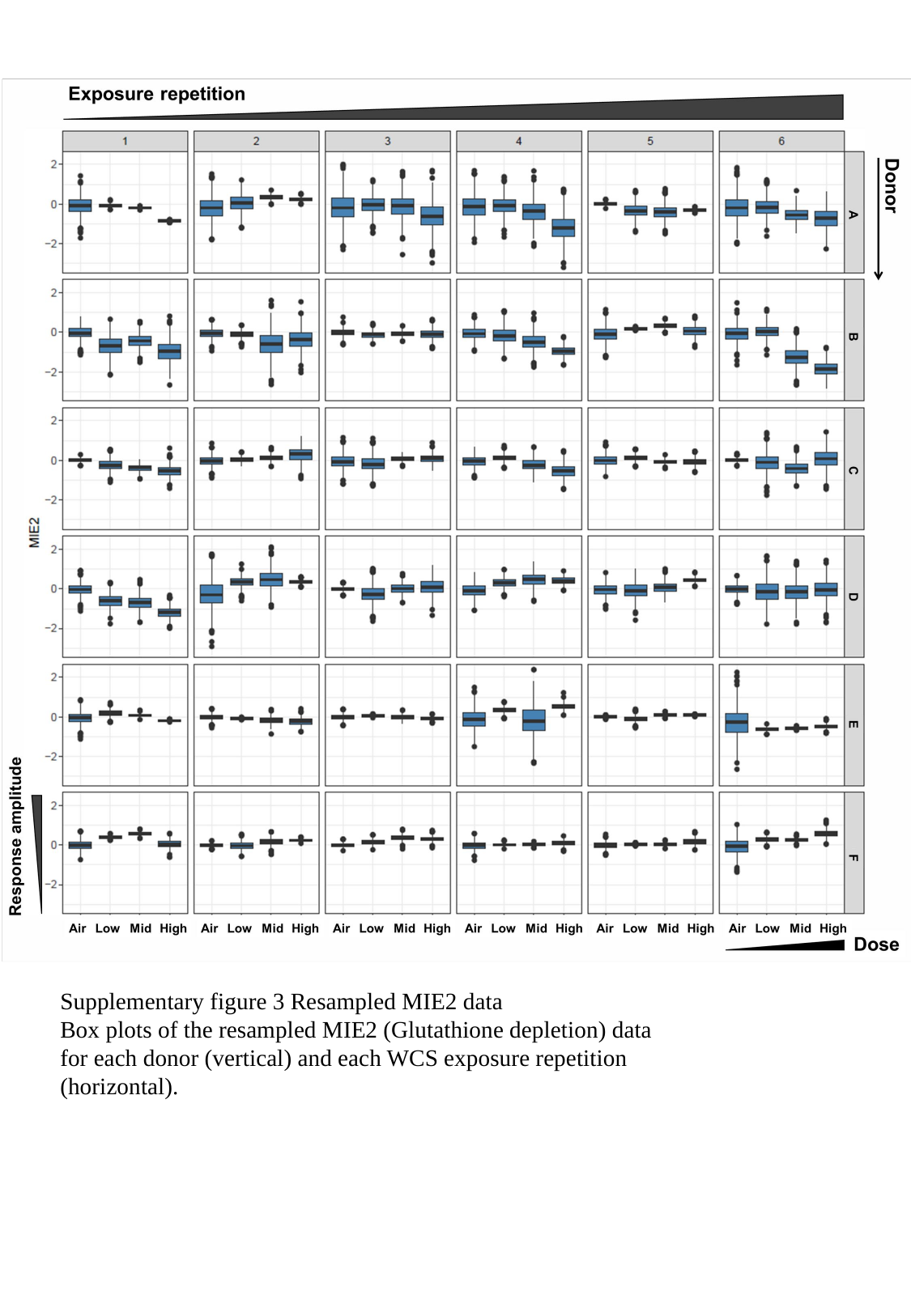

Supplementary figure 3 Resampled MIE2 data
Box plots of the resampled MIE2 (Glutathione depletion) data for each donor (vertical) and each WCS exposure repetition (horizontal).

## Slide 4
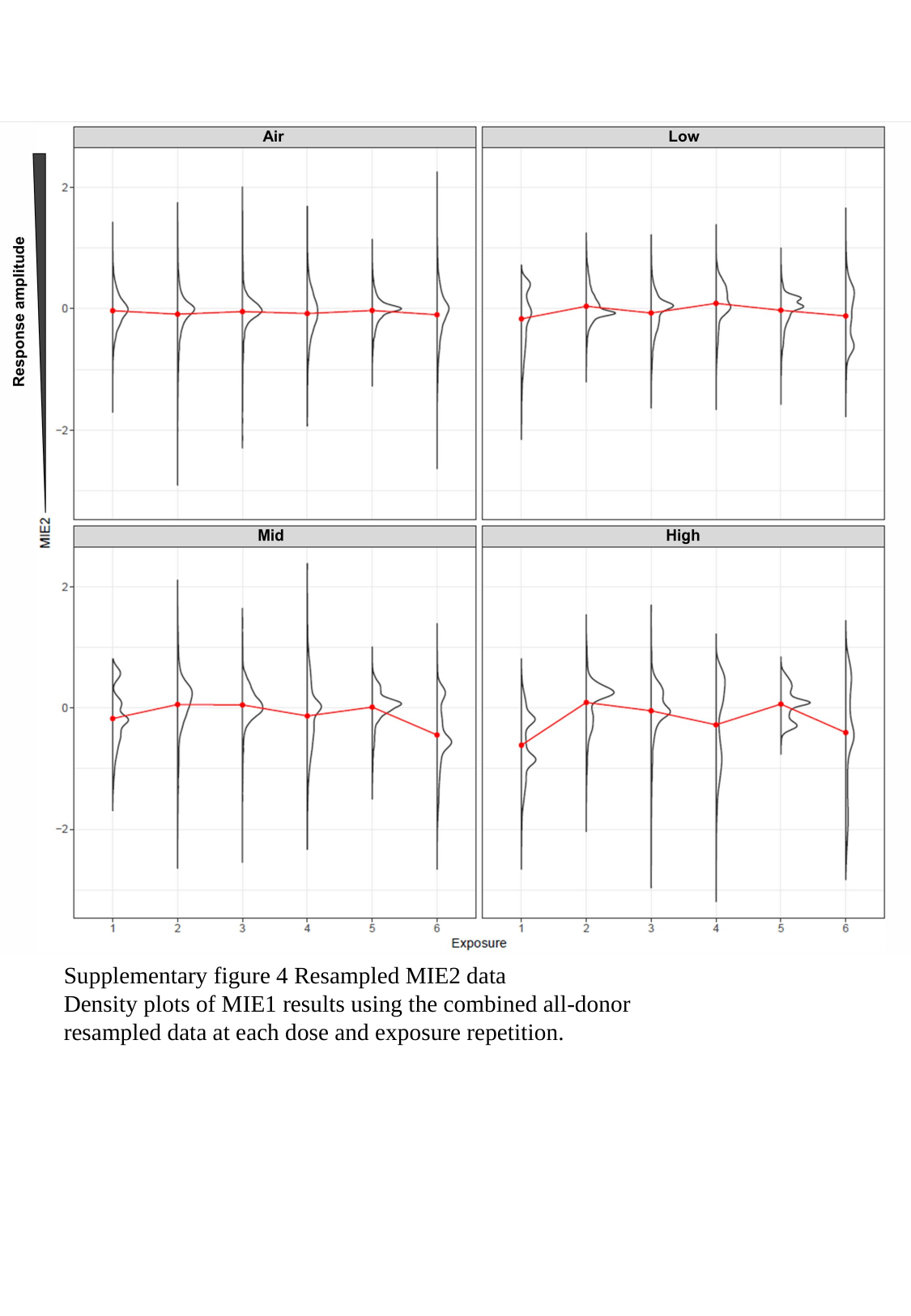

Supplementary figure 4 Resampled MIE2 data
Density plots of MIE1 results using the combined all-donor resampled data at each dose and exposure repetition.

## Slide 5
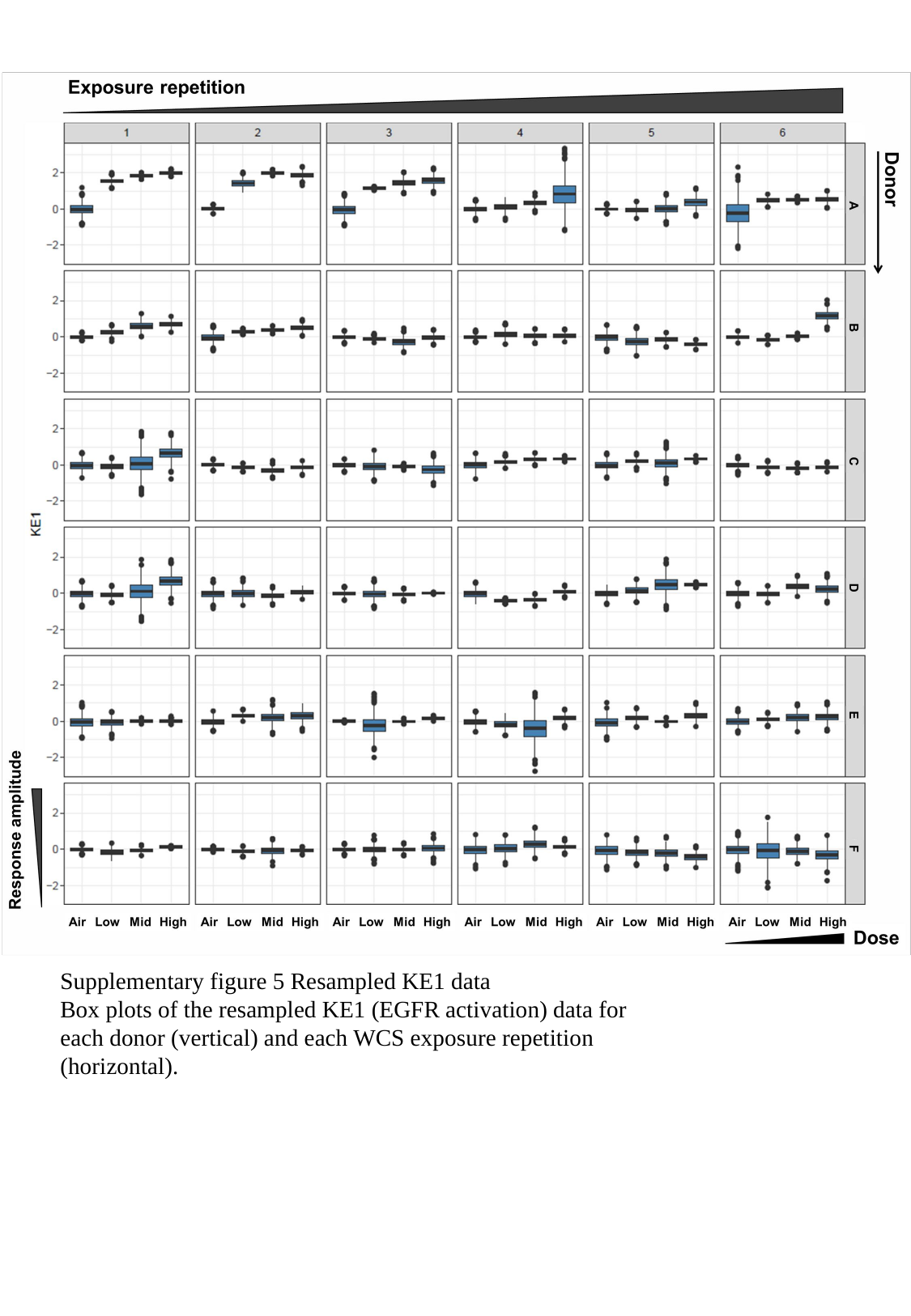

Supplementary figure 5 Resampled KE1 data
Box plots of the resampled KE1 (EGFR activation) data for each donor (vertical) and each WCS exposure repetition (horizontal).

## Slide 6
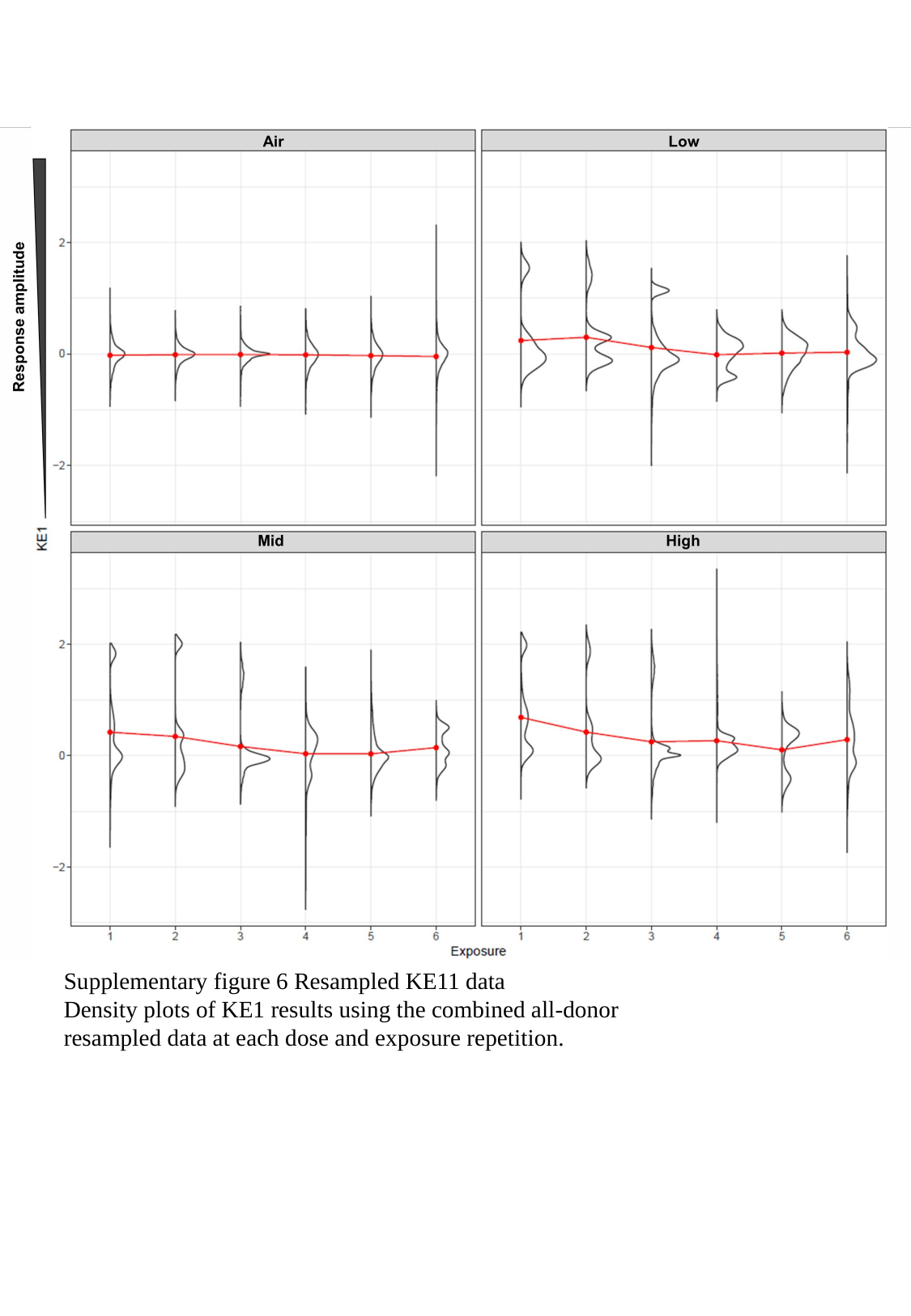

Supplementary figure 6 Resampled KE11 data
Density plots of KE1 results using the combined all-donor resampled data at each dose and exposure repetition.

## Slide 7
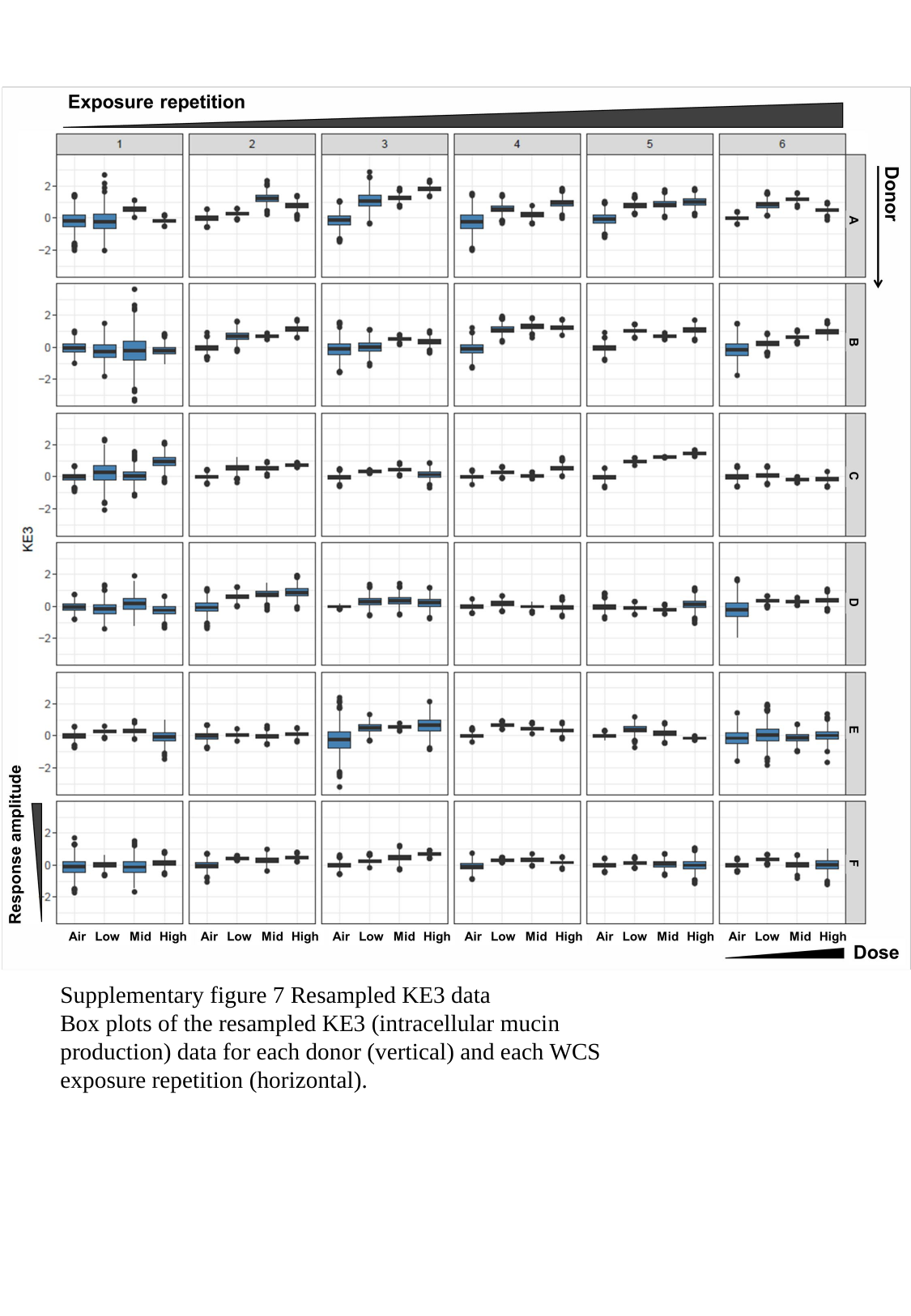

Supplementary figure 7 Resampled KE3 data
Box plots of the resampled KE3 (intracellular mucin production) data for each donor (vertical) and each WCS exposure repetition (horizontal).

## Slide 8
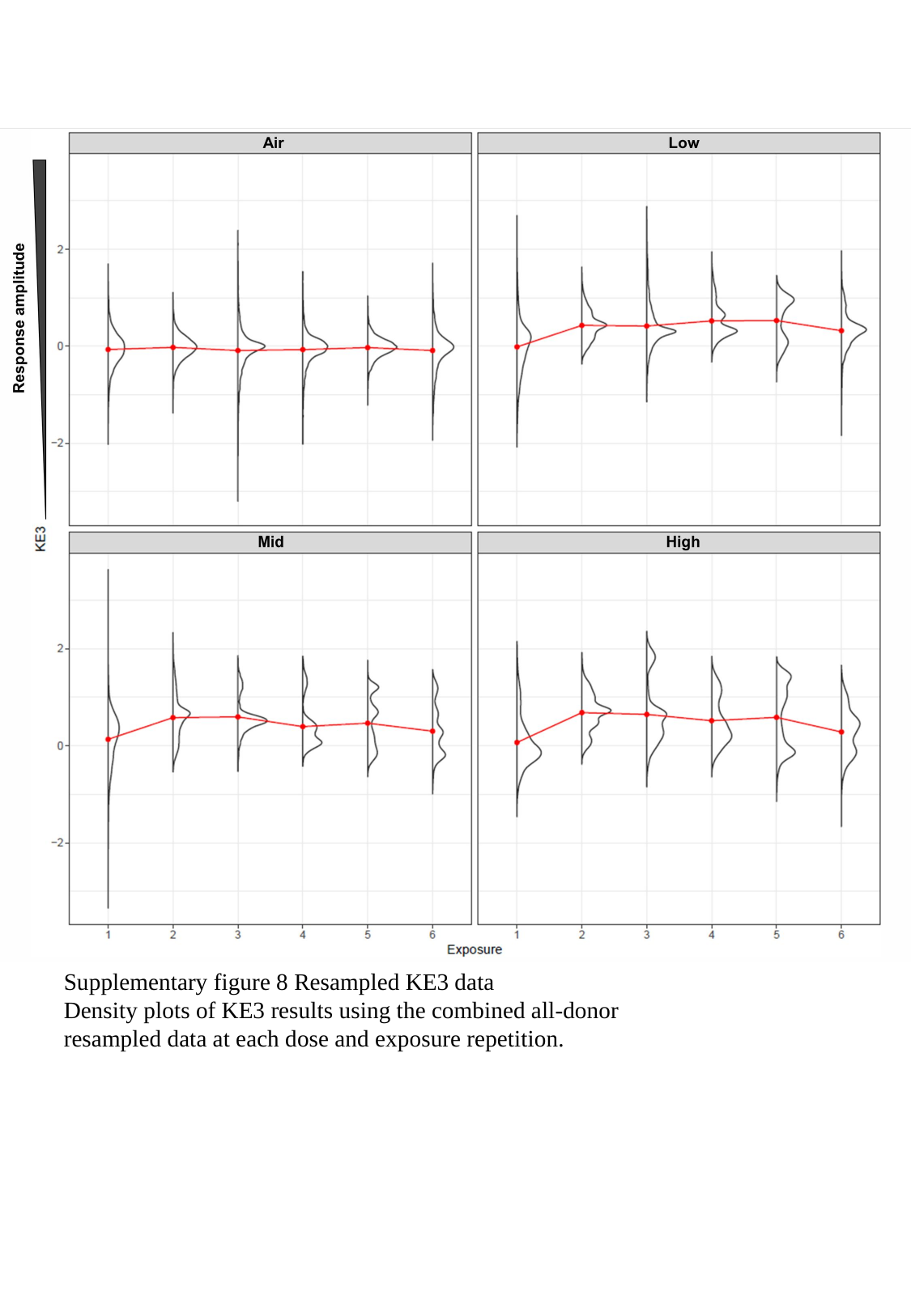

Supplementary figure 8 Resampled KE3 data
Density plots of KE3 results using the combined all-donor resampled data at each dose and exposure repetition.

## Slide 9
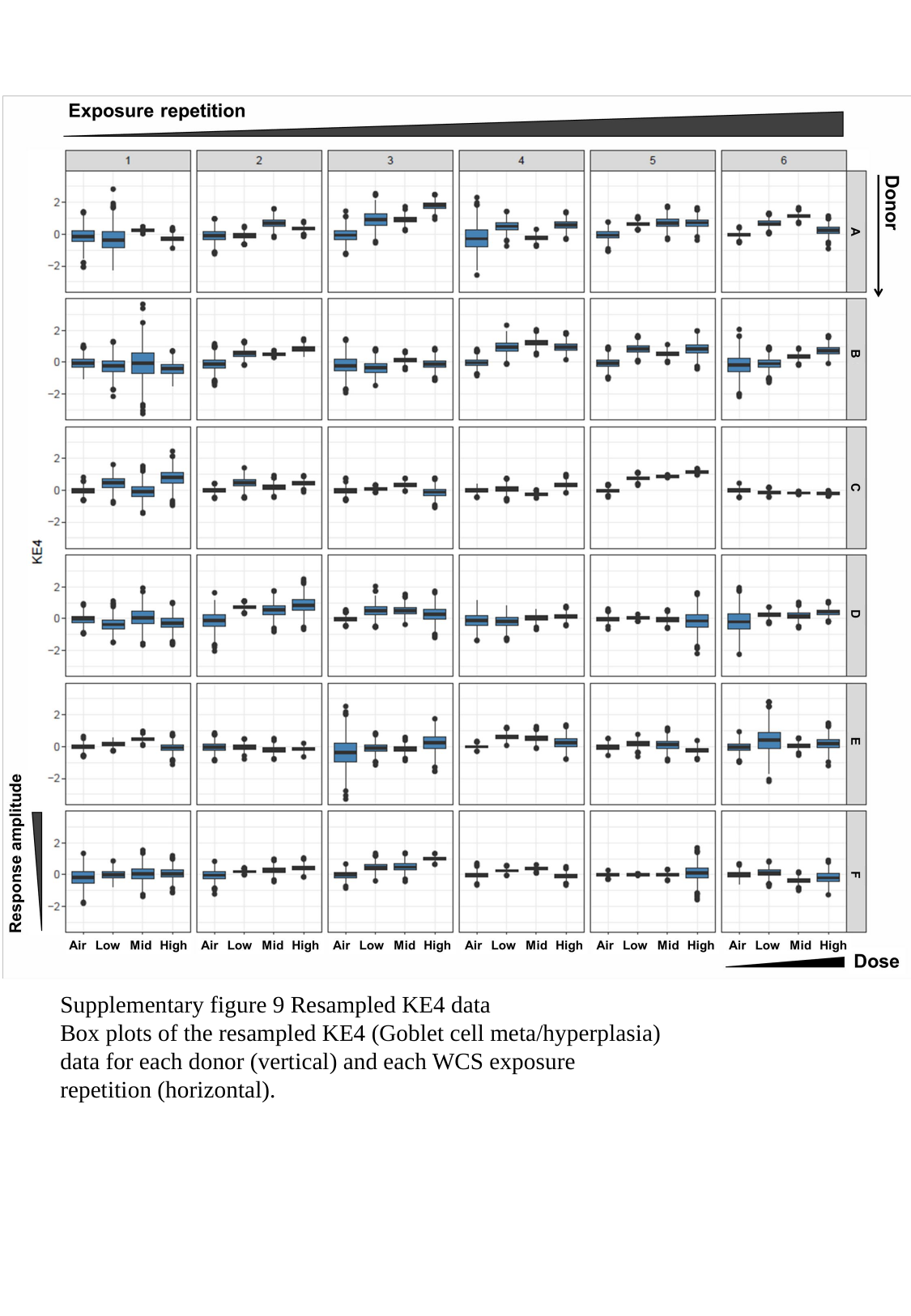

Supplementary figure 9 Resampled KE4 data
Box plots of the resampled KE4 (Goblet cell meta/hyperplasia) data for each donor (vertical) and each WCS exposure repetition (horizontal).

## Slide 10
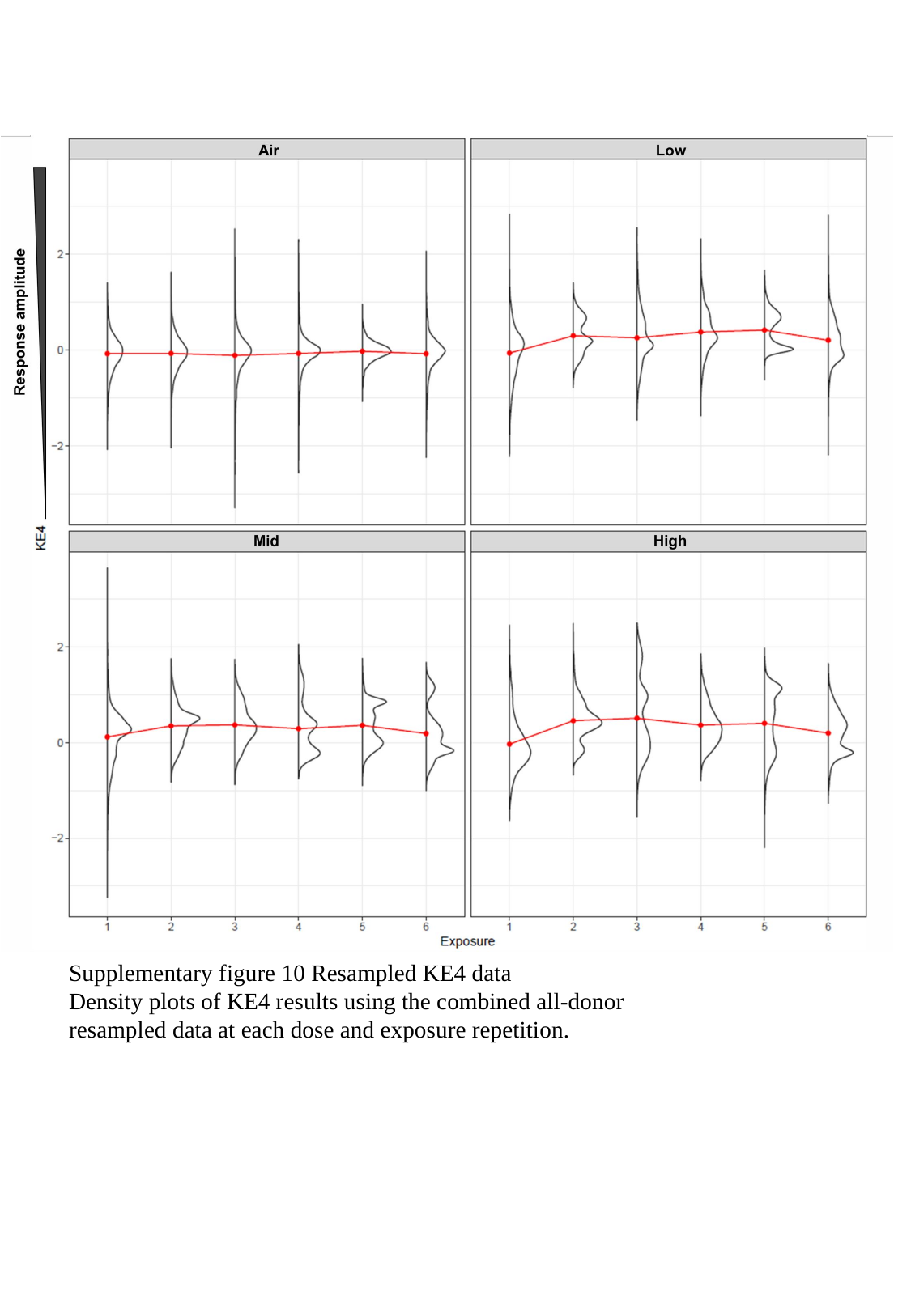

Supplementary figure 10 Resampled KE4 data
Density plots of KE4 results using the combined all-donor resampled data at each dose and exposure repetition.

## Slide 11
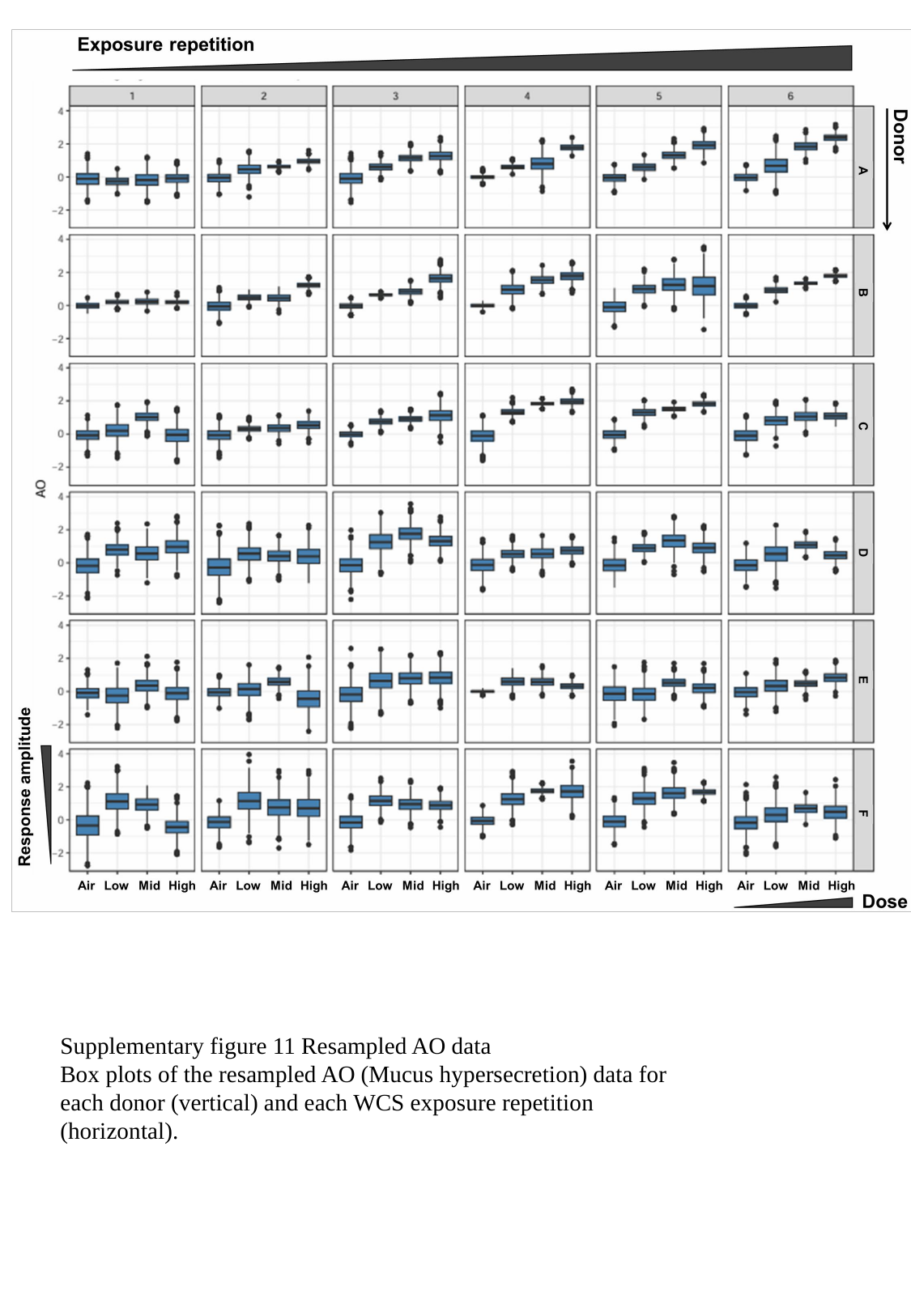

Supplementary figure 11 Resampled AO data
Box plots of the resampled AO (Mucus hypersecretion) data for each donor (vertical) and each WCS exposure repetition (horizontal).

## Slide 12
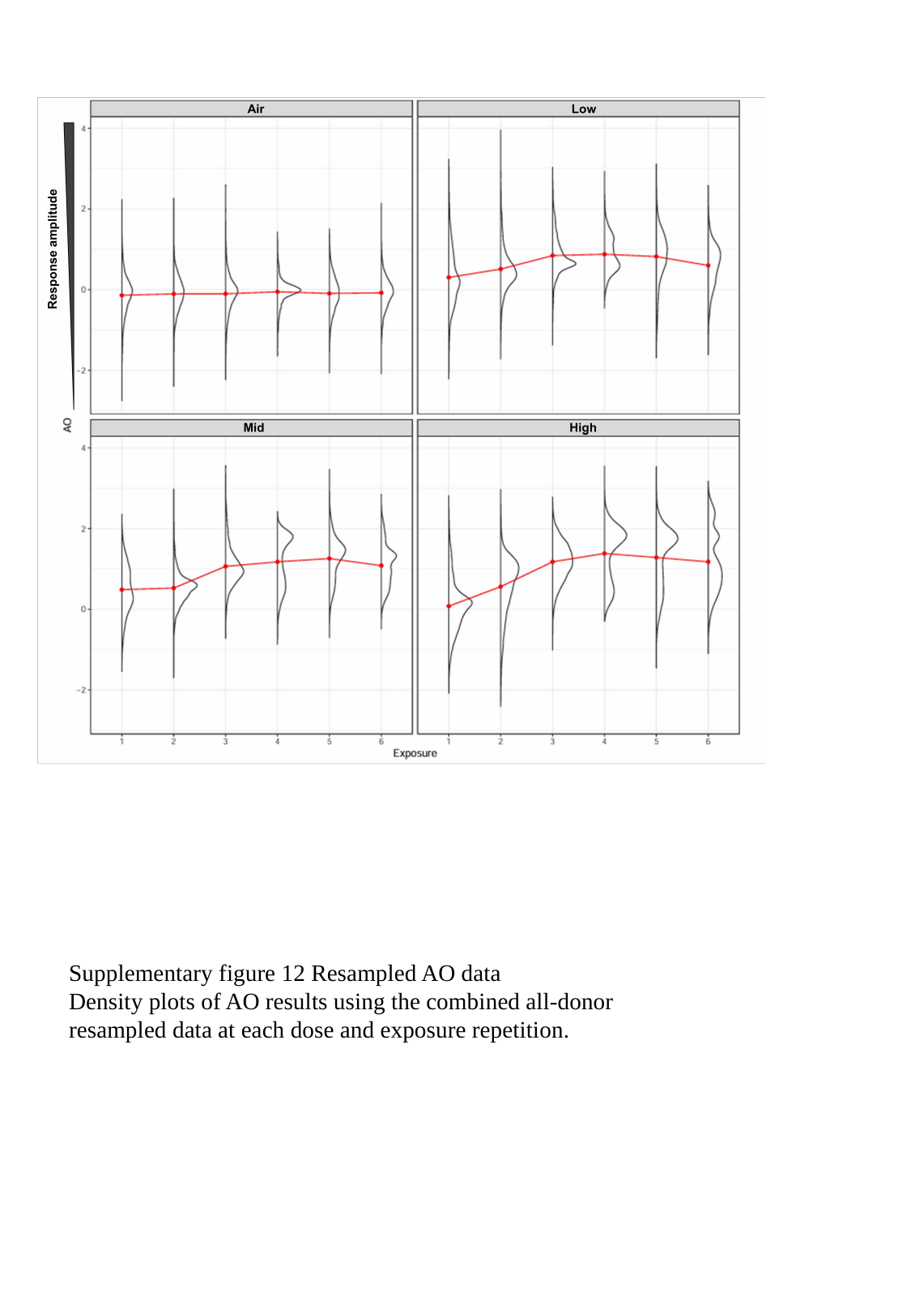

Supplementary figure 12 Resampled AO data
Density plots of AO results using the combined all-donor resampled data at each dose and exposure repetition.

## Slide 13
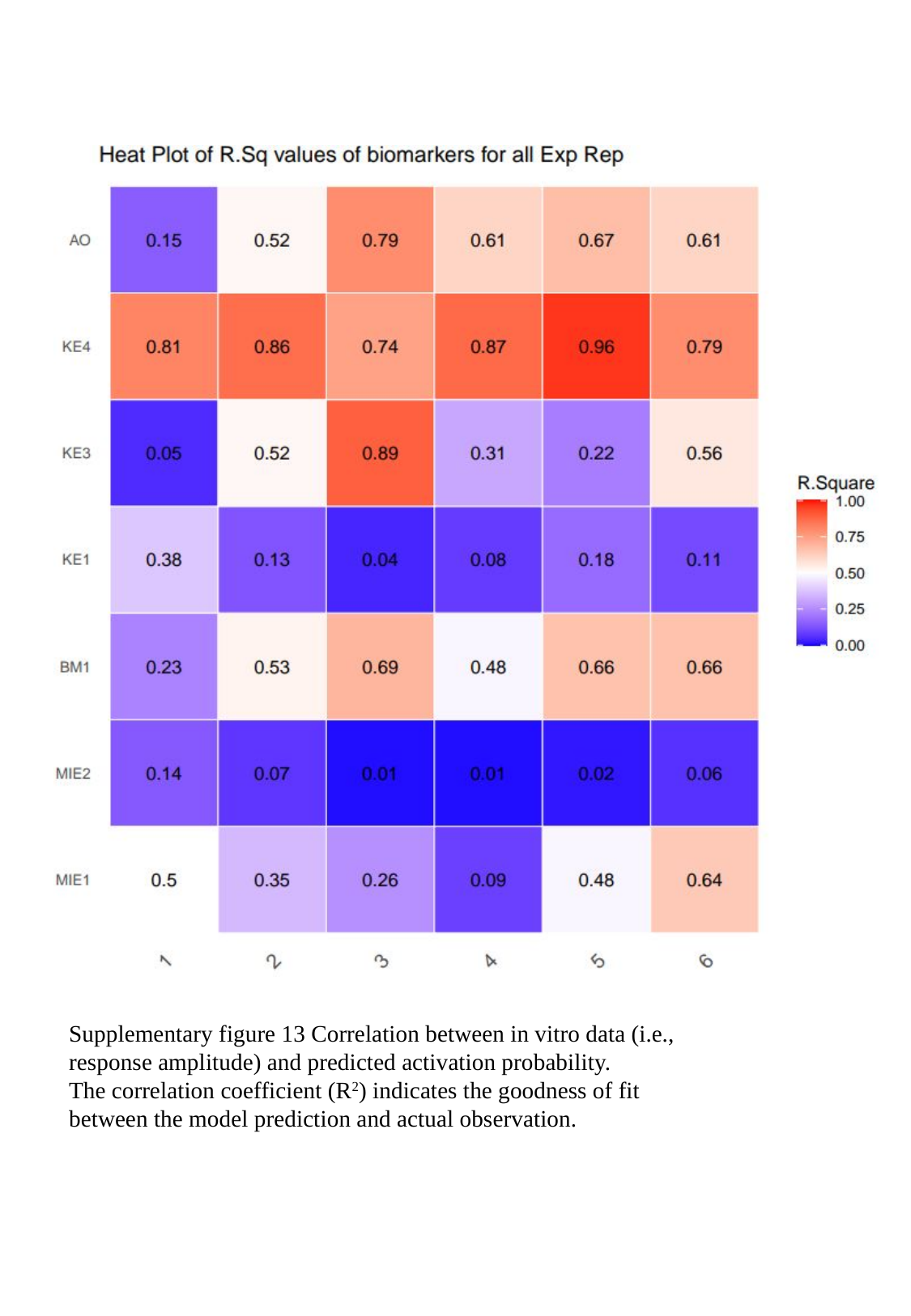

Supplementary figure 13 Correlation between in vitro data (i.e., response amplitude) and predicted activation probability.
The correlation coefficient (R2) indicates the goodness of fit between the model prediction and actual observation.
